# Bi-layer sandwich film for antibacterial catheters

**DOI:** 10.3762/bjnano.8.199

**Published:** 2017-09-22

**Authors:** Gerhard Franz, Florian Schamberger, Hamideh Heidari Zare, Sara Felicitas Bröskamp, Dieter Jocham

**Affiliations:** 1 Munich University of Applied Sciences, Munich, D-80335, Bavaria, Germany; 2 Plasma Parylene Systems, Pang, D-83026, Bavaria, Germany; 3 University Hospital of Schleswig Holstein at Lübeck, Lübeck, D-23538, Schleswig-Holstein, Germany

**Keywords:** catheters, chemical vapor deposition, parylene, sandwich films

## Abstract

**Background:** Approximately one quarter of all nosocomial infections can be attributed to the urinary tract. The infections are supposed to be mainly caused by implantations of urethral catheters and stents. A new catheter design is introduced with the aim to lower the high number of nosocomial urethral infections. In order to avoid limitations to use, the design is first applied to conventional commercially available balloon catheters.

**Results:** The main feature of the design is a sandwich layer on both sides of the catheter wall, which is composed of a fragmented base layer of silver capped by a thin film of poly(*p*-xylylene). This top layer is mainly designed to release a controlled amount of Ag^+^ ions, which is bactericidal, but not toxic to humans. Simultaneously, the lifetime is prolonged to at least one year. The base layer is electrolessly deposited applying Tollens’ reagens, the cap layer is deposited by using chemical vapor deposition.

**Conclusion:** The three main problems of this process, electroless deposition of a fragmented silver film on the surface of an electrically insulating organic polymer, irreproducible evaporation during heating of the precursor, and exponential decrease of the layer thickness along the capillary, have been solved trough the application of a simple electrochemical reaction and two standard principles of physics: Papin’s pot and the principle of Le Chatelier.

## Introduction

In 2014, nosocomial infections caused the death of more than 2000 patients in Swiss hospitals. About one quarter of the deaths were due to infections of the urethral tract. Applying this number to Germany with 10 times the size in population, these infections would have caused the death of approximately 5000 hospitalized patients. As the main reason for these infections, the urethral balloon catheters have been identified, which are implanted into the urethra to almost every sixth hospitalized patient, especially those who undergo a surgery [1]. According to Saint et al., catheter-associated urinary tract infection (CAUTI) is the most frequent health care-associated infection in the USA [2].

In the year 2015, a total of 19.2 million patients were hospitalized in Germany, which meant a consumption of more than three million balloon catheters [3]. To this figure, about 350,000 ureteral stents, which are implanted in the ureters between kidneys and bladder to ensure the drainage of urine, have to be added for those patients with even worse illnesses involving also the kidneys. To emphasize the importance of this issue, the federal government of Germany has launched a program in 2015 that addresses some of these topics, including the conduct in intensive care units but also the development of new devices [4].

For the urethral system, huge efforts has been taken to fight these infections at the root. The most simple and most promising vehicles are antibacterial balloon catheters and ureteral stents. The antibacterial coating of these stents should not only prevent the ascend of bacteria into the renal pelvis, but also the formation of encrustations. Bacteria, especially *proteus mirabilis*, release urease, an enzyme that is capable to hydrolyze urea into ammonia, thereby enlarging the pH value. In turn, the formation of inorganic deposits (mainly hydroxyapatite, calcium oxalate and struvite) is promoted [5]. In ureteral stents with their maximum lumen of 1 mm, these deposits can completely block the drainage [6]. In both systems, the initial colonization of bacteria can be fought relatively easily. Once the invasion is permitted and the small islands have grown to a highly structured biofilm with a protecting polysaccharide, the prospects for an effective attack are severely diminished [7].

Because catheterization was a giant step towards a better mobility of patients, combined with a reduced need for care that saves time for the health care personnel, the strategy could only be an improvement of already existing catheters, which means a highly sophisticated wall material. In this course, two main strategies have been evolved: doping the viscous polymer, which will be subsequently formed to an infinite catheter (capillary) by extruding, with an active reagent or developing a coating of the interior and exterior side of the capillary. For the first alternative, it is imperative that the active species must not be coated by the organic polymer, so it remains free to act as antibacterial source.

### The “Erlanger Silberkatheter” (silver catheter of Erlangen)

The “Erlanger Silberkatheter” was described by Guggenbichler et al. in the 1990’s [8–9]. Remembering that for more than 2000 years, the antibacterial impact of silver has been known, and that bacteria have developed antibiotic resistance against several antibiotics, but not against silver, he also emphasized that silver is also known for its oligodynamic impact, because it can interact with a bacterium in a versatile way. In 2003, however, it was communicated by Silver et al. that they had detected a bacterial resistance against silver by molecular genetics [10]. The impact is not predictable, because this was a single result of research.

**Figure 1:**
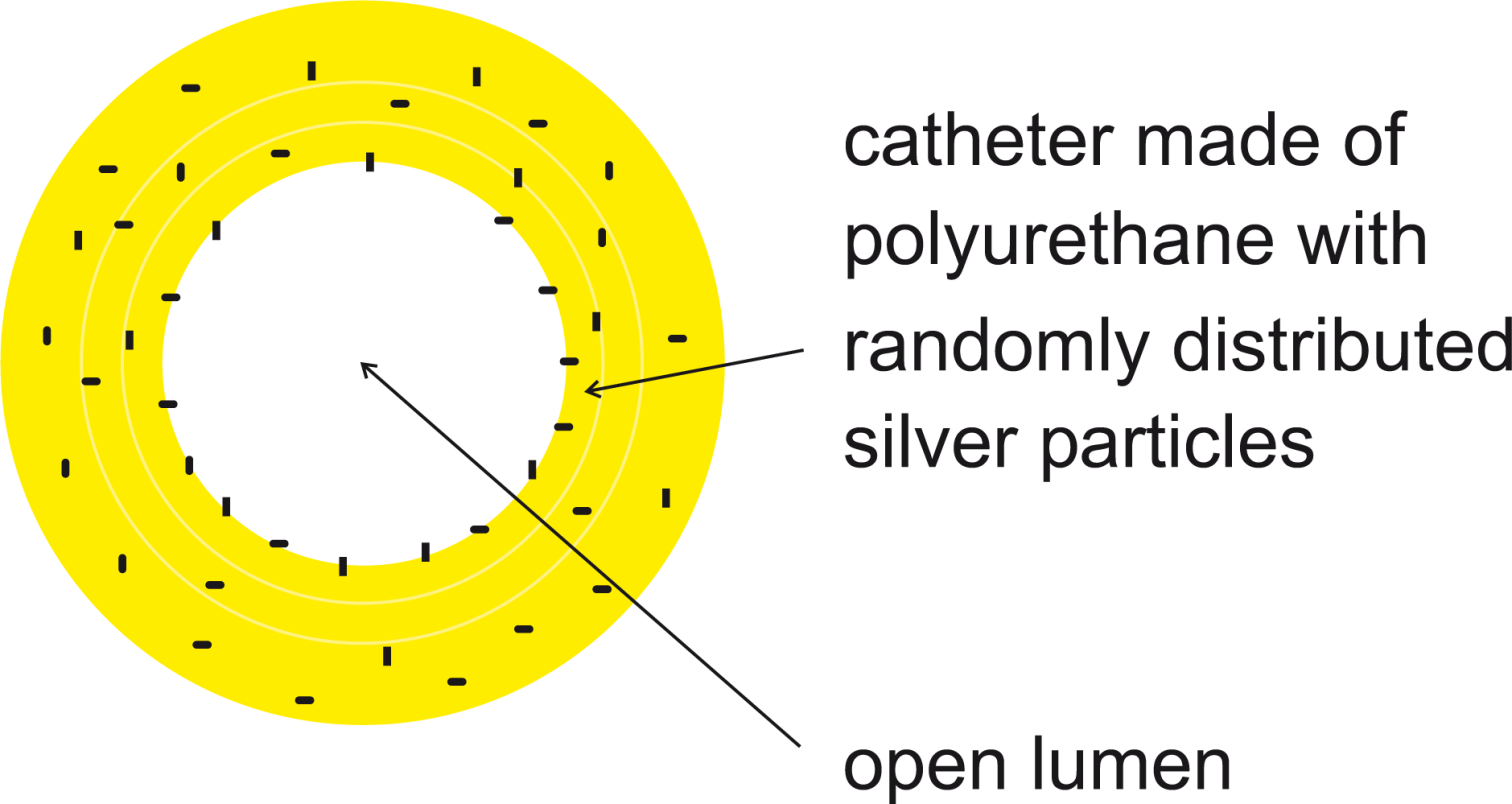
Cross section of Guggenbichler’s “Erlanger Silberkatheter”.

Briefly, plates of organic polymers (polyurethane) were coated with evaporated silver films, hatched into small pieces and added to the highly viscous pastry that was subjected to the extruding process, yielding randomly distributed silver particles in the wall of the capillary (Figure 1). The charm of this technique is its simplicity which offers potential for a large production scale, and large numbers are demanded by the market.

Although it was rapturously applauded by an interested public when it was introduced, it was withdrawn from the public after only a short time of use. Evidently, the clinical trials did not fulfil the promises that were fueled by electrochemical analyses around the Münstedt group [11–12]. The reason is still unclear [13].

### Drug-release catheters

**Figure 2:**
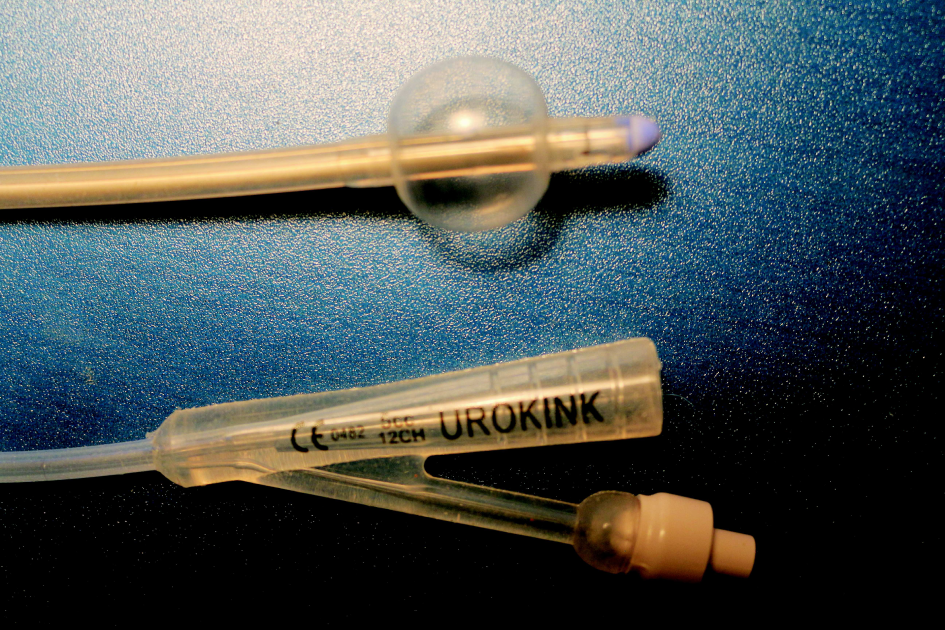
Terminals of a typical balloon catheter. The pipe that connects the terminals has a typical length of 30 cm. After implantation, the blown-up balloon will stick in the urinal bladder (top).

All other trials can be subsumed under drug-release catheters being at least bacteriostatic or even bactericidal. The first trials consisted of dipping catheters into a solution of an antibiotic drug (e.g., ciprofloxacin [14]) and subsequent drying of the solvent. Although, by this simple technique with its variant of impregnating the surface, the idea of drug-releasing devices could be realized, it was prone to generate local concentrations above a tolerable level for adjacent human cells. Since no protecting layer was deposited on top of these deposits, only short-term applications were possible. Therefore, the inorganic alternative silver was proposed again, but now as silver coating [7,15]. This deposit dissolves with a lower time constant, thereby reducing the toxic potential combined with longer lifetime. However, the catheters were coated only on their external skin, and again, no protecting layer was deposited. It should be mentioned that all these alternative tracks are based on conventional catheters, which are modified in various ways and methods. Such a currently commercially available catheter is also our substrate (Figure 2).

#### Deficits and our answers

The above mentioned concepts do not address these deficits:

1. The exterior side of the catheter has intimate contact to the urethra along its whole length. Therefore, it is most likely that contamination with bacteria only happens during its implantation. Depositing an antibacterial layer only on the exterior side of the capillary neglects the fact that bacteria mainly ascend through the interior of the catheter.

2. Ag and its ions are well known as antibacterial reagent, but also to precipitate with Cl^−^ ions to AgCl. Since urine is a 0.1 M solution of sodium chloride, silver ions could never work in the intended manner.

3. Simple deposition techniques, such as impregnation and dipping, do not generate a film that steadily sticks on the substrate. Other methods, such as sputtering and evaporation of silver, only affect the exterior of the catheter.

4. In addition to its antibacterial potential, silver is also toxic.

5. The whole film may not deteriorate the qualities of conventional catheters, in particular, it must remain biocompatible with materials that are admitted by the FDA regulations.

Our answers to these challenges are:

1. We consider necessary a double-sided coating for optimum impact, irrespective of whether the catheter is utilized as urethral balloon catheter or as ureteral stent. Due to the high aspect ratio of the catheters (20 to 30 cm in length at with a small lumen of maximal 1 or 3 mm), the only technique to achieve a double-sided coating is chemical vapor deposition (CVD). A homogeneous film would mean co-deposition with at least two molecules, one to build up the film and one medical drug. To act as vapor, this drug has to be evaporated. None of the commonly used organic molecules (antibiotica, heparine, gendine [16–18]) is sufficiently stable to withstand this process. Only an inorganic reagent, i.e., silver or copper, could be used as metallorganic compound. Hence, a homogeneous coating is almost impossible, and the best solution would be a sandwich system of at least two sublayers.

2. Especially for urine, silver can be used as antibacterial reagent. Ag^+^ ions are easily precipitated by Cl^−^ ions. The solubility product is 10^−10^ mol^2^/L^2^. Urine contains approximately 0.1 M of Cl^−^. Silver and silver ions can only be used because urine also contains urease and urea, which generate ammonia, NH_3_. Ammonia is responsible for a successful application of the antibacterial coating, because it forms the very stable complex [Ag(NH_3_)_2_]^+^, which dissolves a possible precipitate of AgCl [19].

3. Among the various deposition techniques, chemical vapor deposition (CVD) is known for its outstanding conformal coatings, in particular on three-dimensional substrates. Because the substrates discussed here are unstable at high temperatures, no inorganic films can be deposited.

4. Silver is known to act as an effective oligodynamic antibacterial reagent with almost no deficiencies, in particular an ineffectiveness against several bacteria that have developed a resistance against this drug. However, its toxic behavior is also well known. Unfortunately, systematic investigations referring to toxicity and long-term exposition are rare. In 1996, the U.S. Environmental Protection Agency (EPA) published values for the long-term oral reference dose (RfD) [20–21]. These values are based on the assumption that certain illnesses, such as necrosis are triggered by silver ions, but only for concentrations beyond a certain threshold value. These values are explicitly denoted as “estimated” and are given with an uncertainty of approximately one order of magnitude (averaged for all human beings of mean age). For argyria, the RfD value was stated to 5 μg/kg/day, referring to a value that was communicated by Gaul and Staud in 1935 [22–23]. Later on, it evidently became more difficult to work on this topic.Argyria is mostly developed by persons who extensively incorporate colloidal silver [24]. Colloidal silver can be prepared by electrolytical or chemical reduction of a silver salt solution and consists of positively charged silver clusters exhibiting a diameter of typically between 5 and 15 nm and, containing approx. 10^3^ to 10^9^ atoms/cluster. From the generation process, it is evident that the clusters mainly consist of atoms, the residual ions are responsible for keeping the clusters apart, thereby suppressing the aggregation to larger units. The ions fight the bacteria in a multifold manner and are evidently replenished from the cluster after having reacted. Finally, toxicity in the uriniferous system is different from oral ingestion or intravenous injections.

5. Among the thousands of possible organic materials, just a few remain fulfilling the requirements of biocompatibility and of the FDA regulations. For the CVD deposition we chose poly(*p*-xylylene) N, PPX-N or parylene N (N denotes an unsubstituted benzene ring, in contrast to, e.g., PPX-C, which denotes a benzene ring with one Cl atom). PPX, a material with teflon-like properties, has been certified as harmless by the FDA.

#### Poly(*p*-xylylene)

PPX is deposited by low-pressure chemical vapor deposition (CVD) in a vacuum apparatus. Chemical vapor deposition differs from physical vapor deposition by the fact that one or more substances are evaporated and undergo a chemical reaction during transport to a surface. The main advantage of CVD is conformal coating even on heavily rugged surfaces, which makes it the perfect candidate even for the interior deposition on narrow tubes.

**Figure 3:**
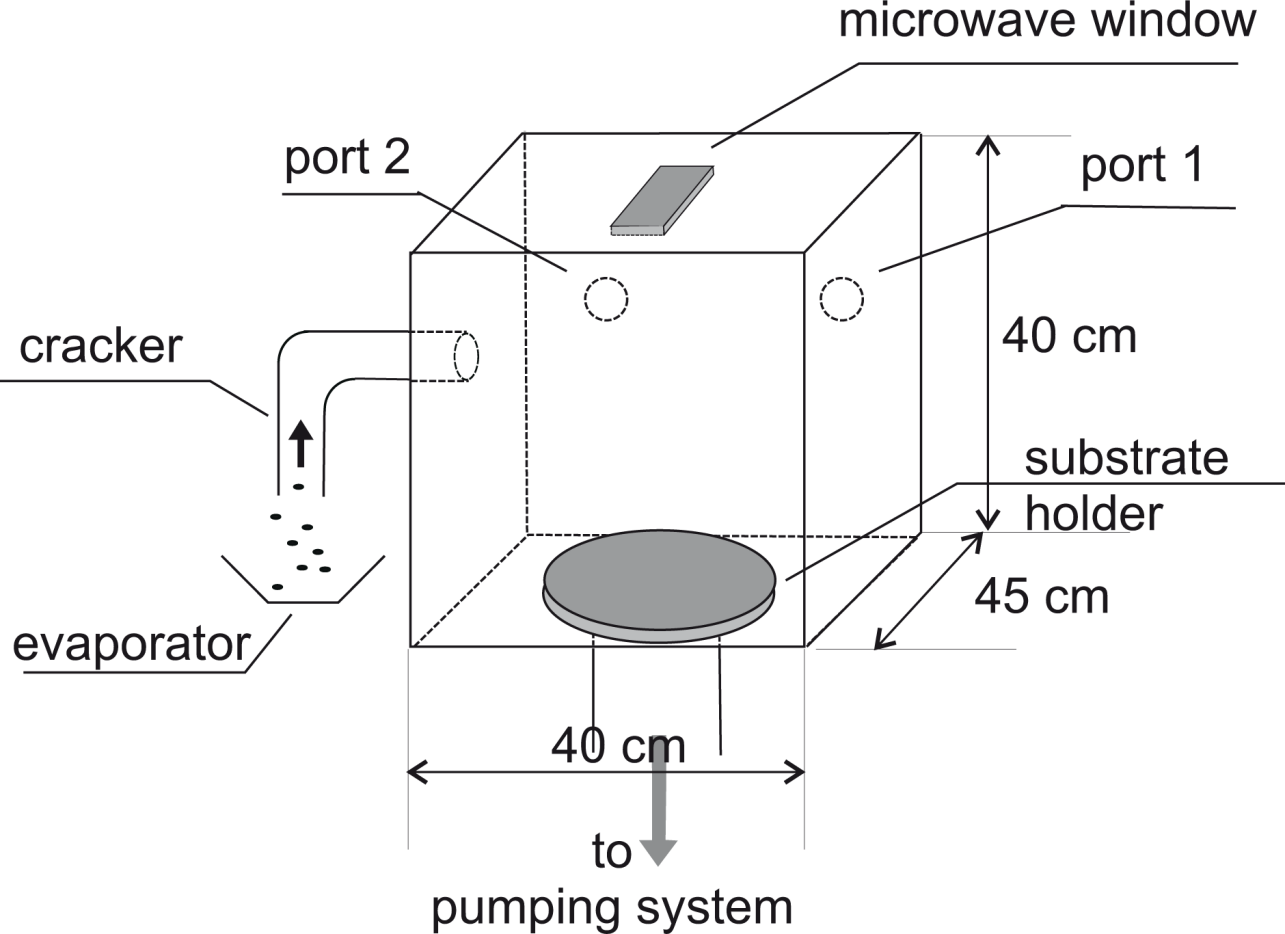
CVD reactor used to deposit PPX. The film-building monomers enter the reactor on the left-hand side and will be pumped out through the annular gap below the circular substrate holder at the bottom. Reprinted with permission from [26], copyright 2013 American Vacuum Society.

Two types of reactors are in use, steady-state reactors and flow reactors. In the first type, a process is started by pressure reduction to a certain level by a vacuum pump. After that the pump is switched off, and the reaction is started [25]). In a flow reactor, the pump is acting during the whole process time, sometimes with reduced pumping power. It is evident that in the first case, the vacuum deteriorates by the presence of inevitable leaks during the process. Especially high-quality layers can be generated only in flow reactors (Figure 3).

**Figure 4:**
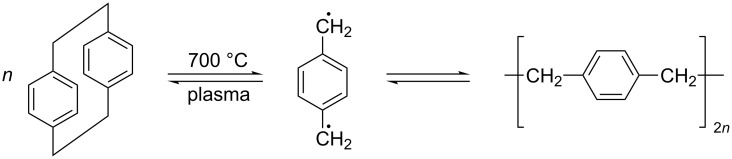
CVD process: The dimeric species di(*p*-xylylene) (DPX, left), which contains two ethyl bridges in each *para*-position, is cracked to form the *p*-xylylene diradical (PX, middle), which forms polymeric chains of poly(*p*-xylylene) (PPX, right) [27]. This reaction can occur either in the gas phase (volume polymerization) or on a cold surface (surface polymerization).

Following Gorham, PPX is deposited by thermally cracking the precursor di(parylene N) (DPX) at 700 °C [27–28] (Figure 4). According to Figure 4, the radical polymerization reaction occurs at the two methylene groups in *para*-position of the benzene ring. This is one of the very rare reactions in organic chemistry with only one reaction route.

#### Design of the sandwich layer

In principle, two layer designs are possible: one homogeneous layer with immersed silver particles, or a sandwich system with at least two layers, one silver depot layer and one protecting top layer. Doping with silver would require copolymerization with a silver-organic compound that has to be decomposed simultaneously. However, silver-organic compounds that can easily be applied are commercially not available. Therefore, fabrication of a homogeneous antibacterial layer is not feasible, and a sandwich system must be developed, which consists of a depot layer of silver capped by a protective layer.

**Figure 5:**
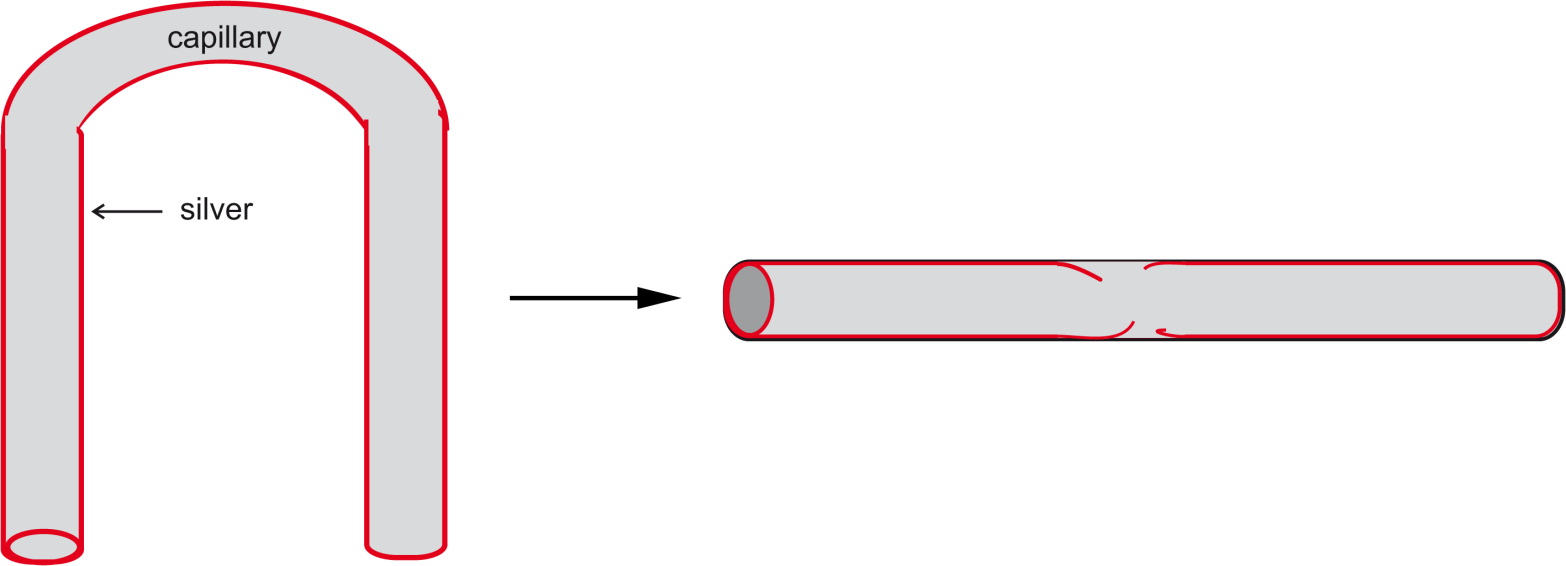
Bending and torsion of the capillary during implantation or usage lead to delamination of the silver-containing layer.

**Silver layer:** Depositing a metallic layer atop a material that is electrically isolating and an organic polymer that is classified as elastomer generates at least two interface problems: (1) How can a film of metallic silver be deposited electrolessly on a surface of an insulating material? and (2) How can the very different Young’s moduli be adapted in such a way that bending and torsion, which are inevitable during the implantation process or during usage, do not cause exfoliation of the silver film? The bendability of the polymer is larger by orders of magnitude than that of the coating. Therefore, a special design has to be applied to avoid cracks and exfoliation (Figure 5).

Our designs are called “zebra stripe pattern” and “leopard skin”. In both cases, only fractions of the total area are coated. Here, we describe the first design.

In the literature, two different coatings are discussed: metallic layers consisting of Ag^0^, and layers containing ionic Ag^+^ salts [10]. Coatings of silver halides are difficult to prepare, the most commonly applied process is impregnation, dipping into a solution or aqueous slurry of a silver salt [17–18,29]. The main issue is the low adhesion of these films. Therefore, the deposition of metallic silver has come to the fore. Because the substrate (organic polymer) is electrically insulating, the most common technique, electrolysis, is not applicable. Only an electroless deposition can lead to the intended pattern. We chose Tollens’s reaction to deposit fragmented metallic silver layers, which is described elsewhere [30–31]. The recipe (concentration and reaction conditions) was adopted from the textbook “Organikum” [32]. Briefly, the redox reaction consists of the oxidation of monosaccharides or disaccharides, accompanied by a reduction of silver ions to elementary silver. The silver forms a fine grained, mirror-like deposit, provided the film-building Ag^+^ can form complex ions, preventing the coagulation to large grains. To classify this method in terms of nanotechnology, it is a bottom-up technique. Layer growth from zero level passes through several stages until the single grains have built a coherent film. This process is visualized with scanning electron microscopy (SEM).

**PPX layer:** The cap layer must meet at least two requirements. First, it must protect the silver from unintended corrosion. Second, it must ensure a certain release rate of the reagent Ag^+^, which is below the toxic level but sufficiently high to fight bacteria, germs and fungi successfully. The minimum inhibitory concentration (MIC) must be exceeded. For this purpose, the coating must be conformal. This means it needs to exhibit a certain constant porosity along the capillary, i.e., a certain and reliable thickness along the catheter, irrespective of whether the surface of the bottom layer is bent or parallel to the wall.

**Figure 6:**
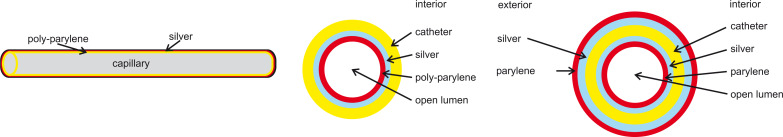
Left: The challenge of coating the interior wall of a closed-end capillary with an aspect ratio of up to 1:100 (diameter/length) with a sandwich system consisting of two layers with a thickness of significantly less than 1 μm (middle). Right: Cross section of the complete system, interior wall and surface of the capillary are coated.

Since the exterior wall surface of the catheter is in intimate contact to the urethra, the antibacterial coating must be applied not only to the interior wall of the capillary but to the exterior wall as well. It is evident that fabrication of an even thickness along the interior wall of a closed-end pipe with an aspect ratio of up to 1:100 (diameter/length) is a challenging issue (Figure 6).

The transport of the film-building species (cf. Equation 1) happens through diffusion (random walk), not through convection (flow). Even for molecules that do not form a deposit, a linear density gradient will form. But deposition of film-building molecules will reduce their density in the vapor, thereby decreasing the growth rate of the forming layer.

At first glance, coating of the exterior wall seems to be easier. However, coating with a layer-forming vapor also deliberately reduces the density of the chain-building species, which causes a reduction of the deposition rate from the vapor entrance (Figure 3, top left) to the pumping port (Figure 3, bottom, distance approx. 50 cm). Although between these points, there exists a gradient in density, the compensation does happen by diffusion, not by convective flow.

For an actual pumping speed of 2.7 L/sec and a gas flow of 5 sccm Ar (1 sccm equals 2.7 × 10^19^ molecules per minute under standard conditions STP (0 °C, 1 bar), which results in a pressure of 22 mTorr (3 Pa) in the reactor (*V* = 72 L), a residence time τ of 42 s can be calculated. Compared to the process of diffusion (mean free path λ ≈ 2 mm, cross section σ = 108 Å^2^, diffusion coefficient *D* = 3750 cm^2^/s with a thermal speed of 550 m/sec), the diffusion length Λ is calculated via the equation for the random walk 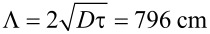. This means diffusion predominates convective flow, and the loss of monomers that will form a polymeric chain via Figure 4 has to be taken into account by setting up the equation of diffusion. Therefore, suspended catheters are expected to be inhomogeneously coated, at least after one shot.

During the deposition of layers within a polymeric tube of a small curvature with a closed end, several challenges have to be faced. First, the number density of the depositing molecules decreases exponentially with penetration depth not only by diffusion but also by deposition losses, which causes a steeply dropping layer thickness. The reaction can occur in the gas phase as well as during or after the process of condensation (physisorption). By diluting the evaporated dimer with argon, the first reaction is suppressed, and the polymeric growth happens only after the condensation of the monomeric species. Irrespective of the route followed, the concentration *c* is described through the diffusion equation reduced by a linear loss term *L* due to the deposition reaction (Equation 1 [33]):

[1]
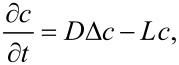


where *c* scales with the partial pressure of the monomeric vapor. Equation 1 is solved by standard methods and is the sum of the two terms in Equation 2

[2]
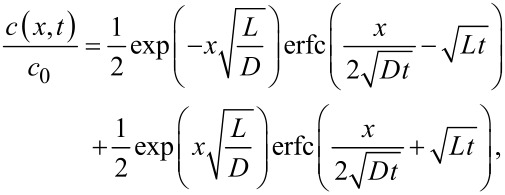


with erfc being the complementary error function.

**Figure 7:**
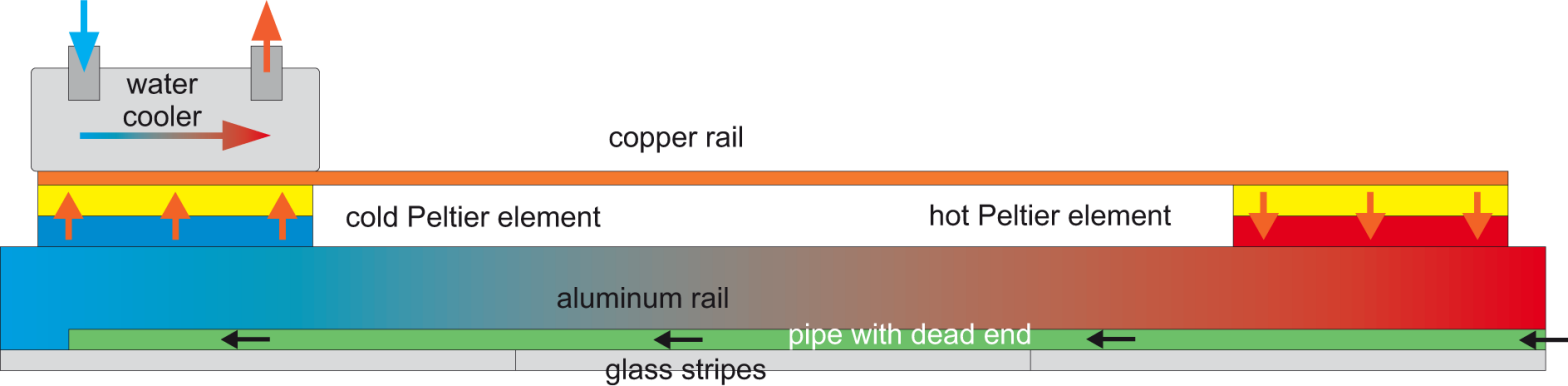
A “temperature seesaw” with several heating or cooling elements can equalize the density gradient that will develop as consequence of diffusion and simultaneous loss of diffusing molecules. Reprinted with permission from [35], copyright 2017 American Institute of Physics.

This issue is the prerequisite for the application of the “temperature seesaw”. Condensation is an exothermic reaction. According to the principle of Le Chatelier, a (phase) equilibrium can be influenced by temperature and pressure. Here, rising the temperature favors the density of the energy-rich side, i.e., the vapor side. Hence, condensation and subsequent polymerization can be forced back or can even be suppressed by an increase of temperature, if the temperature is raised beyond the so-called ceiling temperature [34]. This is the basis for the construction of a temperature seesaw (Figure 7). It consists of a metallic rail with a semi-circular groove cut, which hosts the closed-end capillary. Above this configuration, several Peltier elements (up to five, shown here are two) with thermocouples are located through which a temperature gradient of ±30 °C can be obtained. This gradient is sufficient to counterbalance the density loss of monomeric diradicals along the capillary.

Another challenge is that the degree of porosity depends on the preparation conditions for the protecting layer, in particular of its thickness. The vague expression porosity must be brought into a quantitative relation to the thickness and to the release rate of Ag^+^ ions.

**Figure 8:**
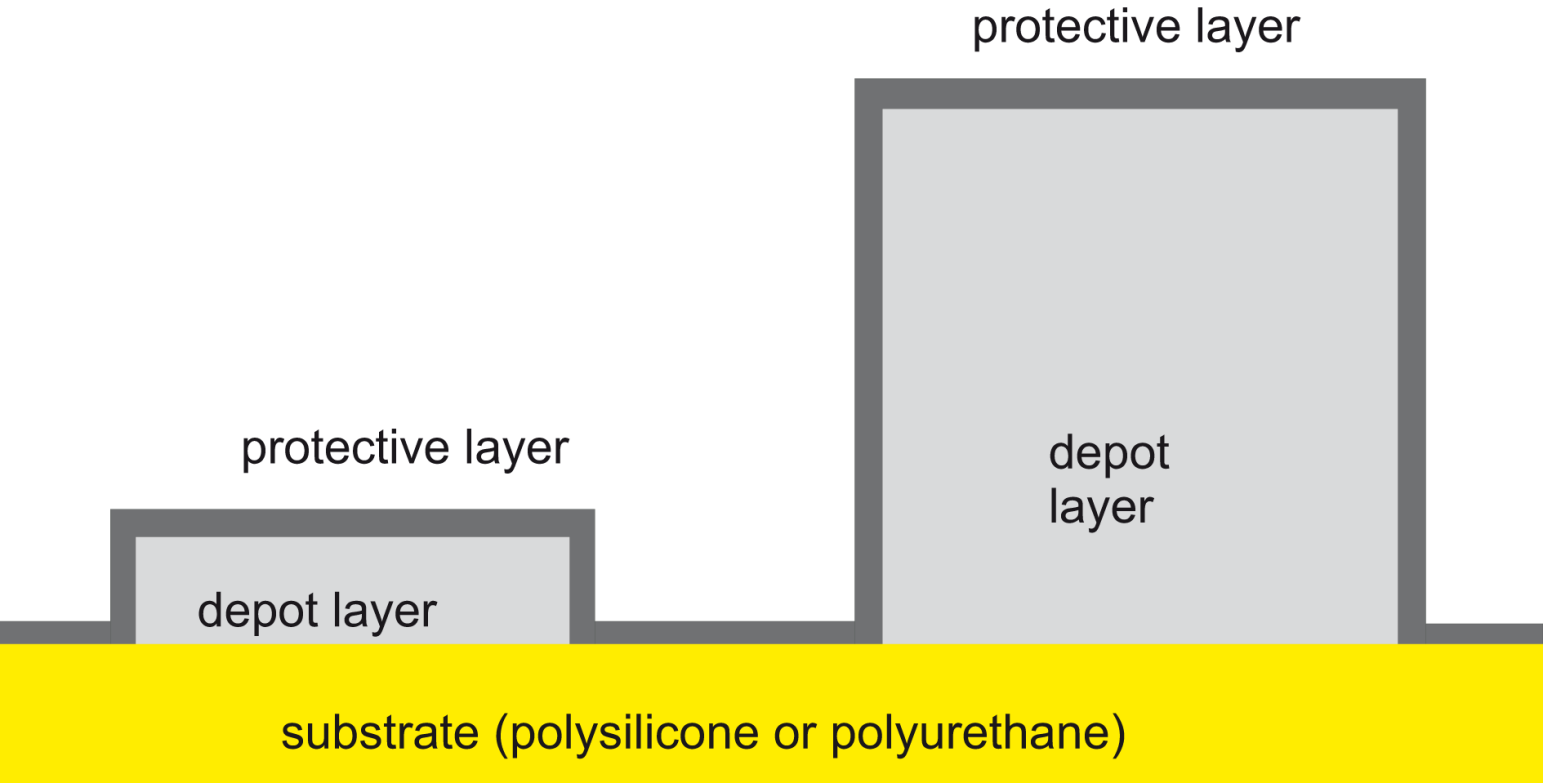
The thickness of the protecting cap layer with its retarding effect determines the thickness of the silver layer with its antibacterial impact. Since the adhesion of the organic polymer atop an organic substrate is far better than the adhesion of silver, the silver depot should consists of grains or stripes, which are encased by an evenly thick layer of PPX.

**Thickness ratio of the sandwich layers:** After having determined the thickness of the cap layer, the thickness of the depot layer has to be fixed. This thickness is a trade-off between durability and adhesion of the sandwich system. It is evident that the adhesion of the silver layer is significantly improved if this layer does not consist of a continuous film on top of the substrate (polysilicone or polyurethane), but if small grains or stripes are encased by the organic polymer. This polymer is deposited directly on the substrate, and the adhesion between an organic substrate and the organic polymer is expected to be far better than the adhesion of silver atop polysilicone or polyurethane. Also, the thickness of the protecting porous layer determines the maximum of the depot layer. For a long durability, the thickness should be as high as possible. For a stable and reliable sandwich system, the thickness of both the layers should be similar (Figure 8).

**Summary:** To coat a catheter with an antibacterial layer, several obstacles have to be overcome:

- the need for biocompatible coating material,
- an antibacterial agent that must be effective against several bacterial strains that have developed resistances against antibiotics,
- a tunable release rate of the agent,
- the deposition of layers with uniform thickness on both sides of the catheter, especially on the interior.

In this paper, the most important steps of the fabrication of urethral catheters with an antibacterial coating are addressed and described:

- the deposition of the silver film,
- the deposition of an organic polymer (PPX) by chemical vapor deposition (CVD),
- the deposition of the interior PPX film,
- and the characterization of the films, in particular the grain size of the silver clusters, the determination of thickness of the PPX film and its influence on the porosity,
- and the influence of the above properties on the release rate of antibacterial Ag^+^ ions.

## Experimental

### Silver film

#### Deposition

The deposition of metallic silver is an electroless reaction in aqueous solution. Silver ions are reduced by a saccharide (glucose or maltose) at elevated temperatures, typically at 70 °C. Of paramount importance is the pre-treatment of the hydrophobic substrates (polyurethane and polysilicon), which can be carried out with an oxygen plasma, either by microwave generation (100 E TechnicsPlasma, Kirchheim, Germany), or by RF generation (PlasmaLab 80, Oxford Plasma Technology, Yatton, UK). Another method involves exposing the substrates (polysilicon) to diluted nitric acid (30%) for approximately 30 min. The latter procedure was preferred, mainly because the process could be controlled visually, and the reproducibility is far better. By this treatment, the nature of the surface is switched from hydrophobic to hydrophilic through the generation of carboxy and hydroxy groups at the surface [36].

**Figure 9:**
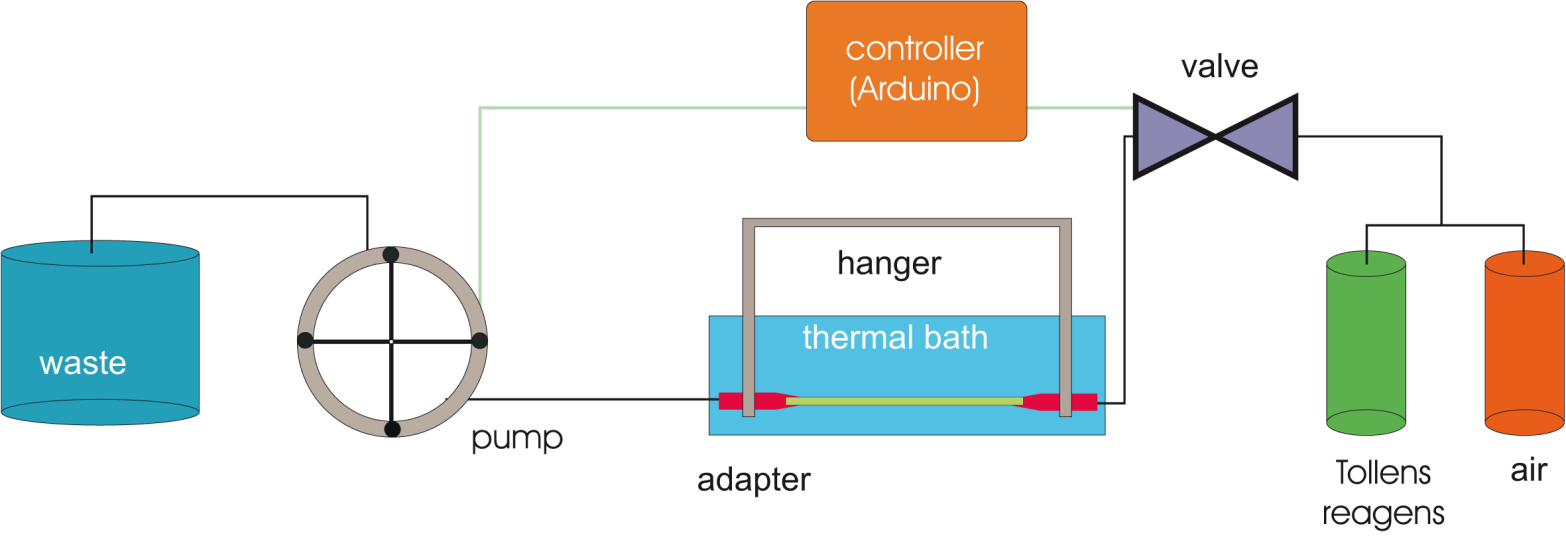
Apparatus for deposition of fragmented silver layers. Reprinted with permission from [19], copyright 2016 American Vacuum Society.

The silver deposition itself was carried out by applying a pneumatic apparatus. The capillary was attached to a small peristaltic pump that draws a mixture of an Ag^+^ solution (AgNO_3_ dissolved in a surplus of aqueous ammonia (type TL, Medorex, Nörten-Hardenberg, Germany), to which a certain amount of a monosaccharide (glucose) or a disaccharide (maltose) is added (denoted as Tollens’ reagent) and air (Figure 9), by which a chain of bubbles is generated, consisting of alternating packages of air and reagent (PD 5101, Heidolph Instruments GmbH &Co. KG, Schwabach, Germany). The volume ratio of these bubbles could be adjusted by a small Arduino controller and was visually inspected (Arduino Proto Sield REV3). The minimum length was the diameter of the capillary. The silver layers were deposited in a water bath (temperature between 70 and 85 °C, mainly at the former value).

#### Analysis

Because the roughness of the polymeric substrates does not allow for an exact measurement of the layer thickness and the growth rate as well as the assessment of the morphology, smooth substrates are required. We deposited a silver layer on microscope glass slides. The layer thickness was measured with mechanical profilometry (α-step, KLA-Tencor, Milpitas, Calif., U.S.A.). The glass slides have the advantage that the exact silver volume of the deposited layers can be measured. For thick layers, the density of the precipitate can be calculated by measuring the different weights before and after deposition.

### PPX layer

#### Deposition

The subsequent deposition of the porous cap layer consisting of PPX is performed with a slightly modified Gorham process [26–27]. In a CVD reactor (Plasma Parylene Systems, Rosenheim, Germany), the dimeric precursor is evaporated at temperatures between 130 and 140 °C and monomerized in the cracking zone at 700 °C (Figure 3). In contrast to a steady-state reactor, this vessel is continuously perfused by the polymer-generating vapor and additional doping gases. Vapor enters through a heated pipe and reacts either in the volume (gas-phase reaction) or on cold surfaces (solid-state reaction) to a polymer by chain-building.

**Figure 10:**
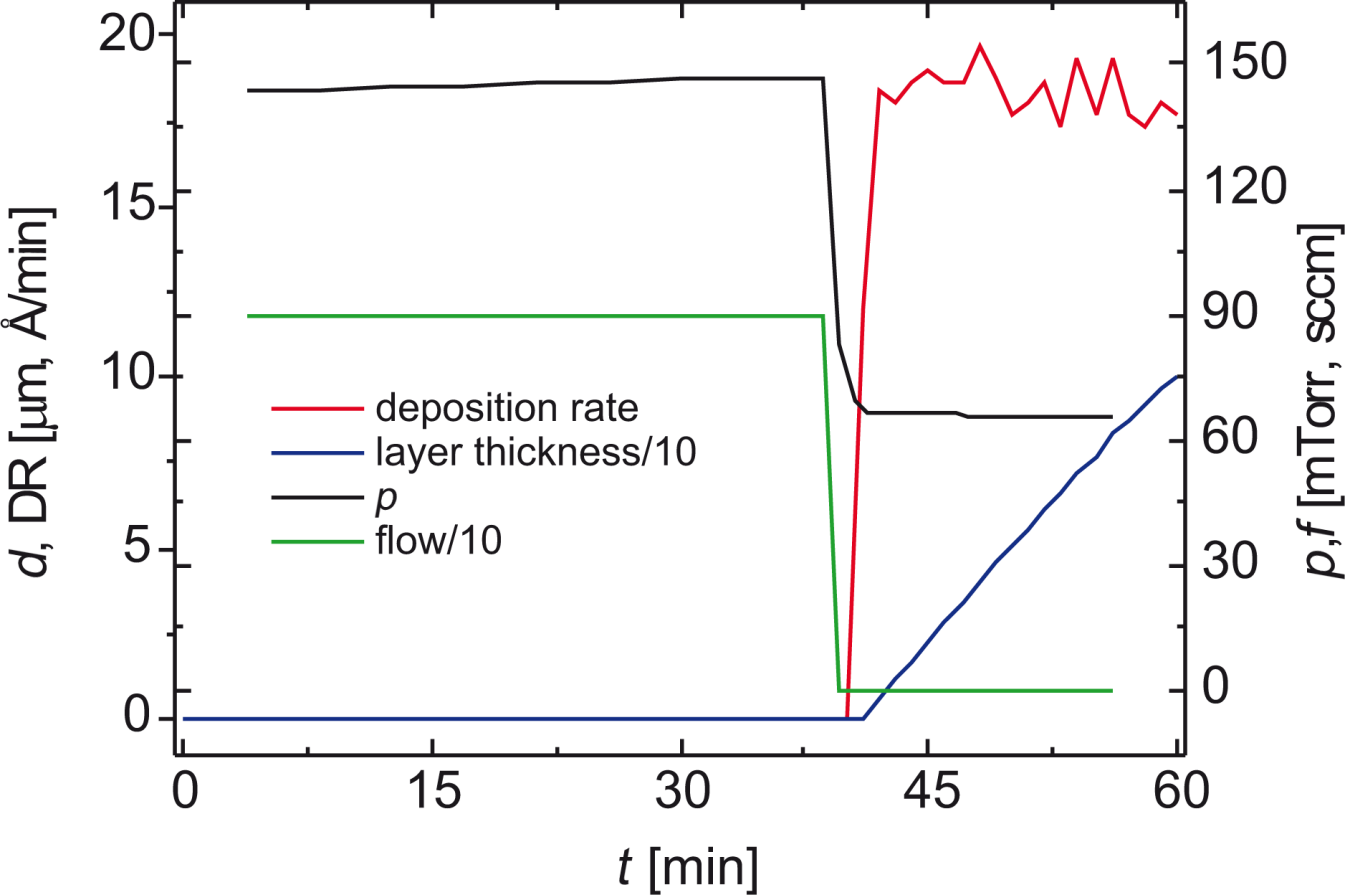
Deposition rate of PPX and counteracting argon pressure during the heating ramp [26,37]. Reprinted with permission from [26], copyright 2013 American Vacuum Society.

To prevent evaporation during the heating ramp (approx. 45 min), which would cause an irreproducible layer thickness, a flow of argon generates a pressure of approximately 300 mTorr (mass flow controller 1179B, MKS GmbH, Munich, Germany). After having reached the evaporation temperature, the argon flow is suddenly lowered, and the deposition starts at a constant rate. At steady state, the entrance flow of the monomer is approximately 9 sccm. The monomer is highly diluted with argon (flows between 2 and 4 sccm), approaching epitaxial conditions, i.e., volume polymerization is suppressed to favor surface polymerization (Figure 10).

For a flow of 10 sccm argon, the pressure would rise to 156 mTorr, and the residence time in the reactor with *V* = 72 L would be 135 s. The velocity of flow in the cracking tube with a cross section of approximately 72 L is 10 cm/s, and the mean free path λ of nitrogen at this pressure would be 0.25 mm. The length *l* of the cracking zone is 30 cm, i.e., a ratio *l*/λ of 1200, which is expected more than sufficient for a complete cleavage of the dimeric precursor DPX. Diluting the organic vapor with an equal amount of inert gas doubles the flow velocity and halves λ.

#### Analysis

**Thickness:** The thickness of the film can easily be evaluated with a mechanic profilometer, but only on a plain hard substrate (glass). Its principle consists of creating an acute step in the film without hurting the substrate. This step is traced with a sharp needle of diamond as in a conventional cartridge of an old-fashioned turntable. Because of the softness of the catheters, no exact measurement is feasible. Therefore, only optical methods can be applied, either in transmission (absorption due to the law of Lambert and Beer) or in reflection (interferometric measurement). In both cases, the capillary has to be sliced to get access to the layer. Because the thickness of the wall is large compared to the thickness of the deposited layer (1,500 μm vs 0.3 μm), this method is hardly applicable. But recording the broad-band reflectance of the capillary will lead to success. We applied the spectrometer F20e from Filmetrics (Unterhaching, Germany) using a light spot with a diameter *d* of 30 μm. For a radius *r* of the capillary of 1.5 mm, the substrate can be regarded plain (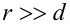). The reflected light is diffracted by a diffraction grating and recorded by a photodiode array. By relating the recorded spectrum of the coated substrate to a previously recorded spectrum of the pure substrate, a background-corrected signal is accessible, which yields thickness and refractive index of the probed layer [35]. Statements of the film thickness refer to the mechanical measurement in the case of porosity and microbiological context, and the optical measurement is used for the homogeneous coating of the interior of the capillary.

**Porosity:** Porosity can be evaluated qualitatively and quantitatively. The qualitative approach comprises visual inspection and scanning of surface areas with atomic force microscopy (AFM). When automatic evaluation procedures are applied, the roughness of the surface can be quantitatively validated. Physical methods involve measurement of the impedance of an incompletely isolating layer on top of an electrode.

Applying AFM, the small areas of the surface can be scanned and evaluated regarding parameters like roughness and porosity. While the first parameter is the standard output of AFM and is displayed as 3D topographical map with mountains and valleys, porosity only regards those valleys that extend to the substrate (holes). To discriminate between valleys and holes, we applied the evaluation software GWYDDION [38], which extracts height profiles *h*_profile_ along a scanned line *l*_scan_, for which we chose the largest possible measurement area (130 μm × 130 μm). We set the threshold for a hole to 90% of the total thickness measured with mechanical profilometry.

**Figure 11:**
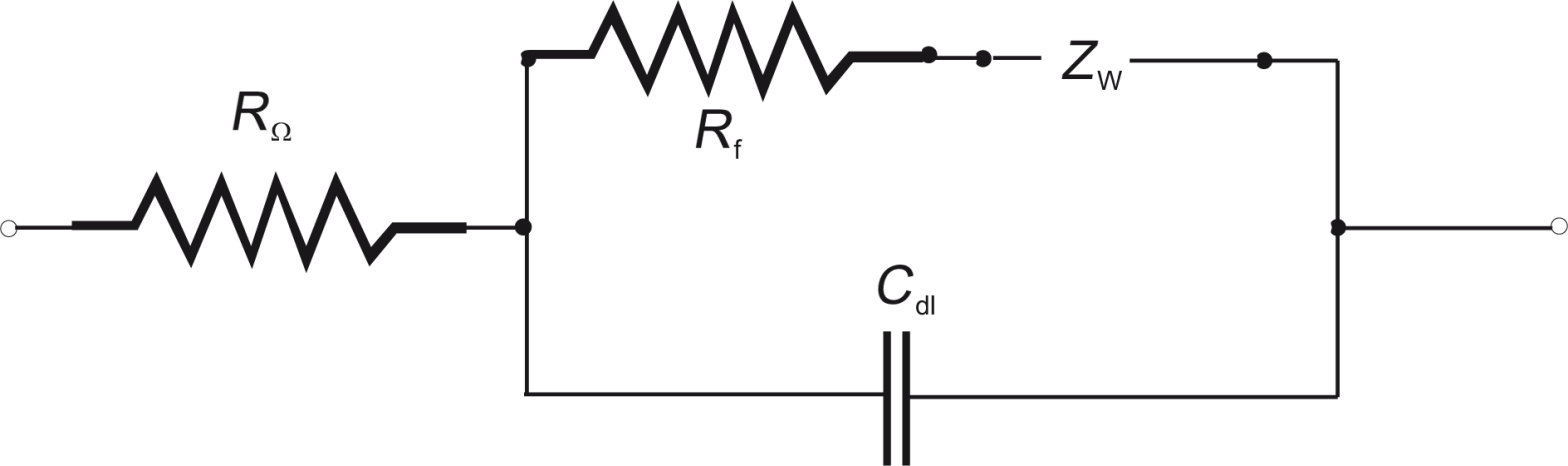
Equivalent circuit of the electrode covered by a porous membrane with capacitance *C*_dl_ and ohmic resistance *R*_f_ in series with the Warburg impedance *Z*_W_ in an aqueous electrolyte (*R*_Ω_). Reprinted with permission from [39], copyright 2012 American Vacuum Society.

Physical inspection comprises measurements that make use of electric currents flowing through the pores and holes of an imperfect dielectric medium. This method is called electrical impedance spectroscopy (EIS) and is a widespread technique to evaluate the quality of coatings quantitatively. In contrast to the first method, larger areas can be easily tested, and a quantitative result is yielded, under the expense of spatial resolution. A simple electrochemical cell, which consists of two electrodes in an electrolyte with defined concentration and DC conductivity, is used as basis. One electrode, the device under test, is coated with a porous layer of a dielectric medium, here PPX, which would yield an infinite DC resistance for perfect coverage. Increasing the frequency of the voltage would only generate a displacement current. For a porous layer, however, ions are attracted by the potential and generate a conduction current. This additional current scales inversely with the square root of the applied frequency, because the ionic current depends on the distance the ions can travel during their attractive half period. Hence, the total resistance of this electrode is the sum of ohmic resistance of the PPX film, *R*_f_ and the frequency-dependent Warburg resistance, *Z*_W_, because *R*_f_ is in series with *Z*_W_. Parallel to this series resistance is the capacitance of the electrochemical double layer, *C*_dl_. *R*_Ω_ is the ohmic resistance of the solution of the electrolyte (Figure 11).

**Figure 12:**
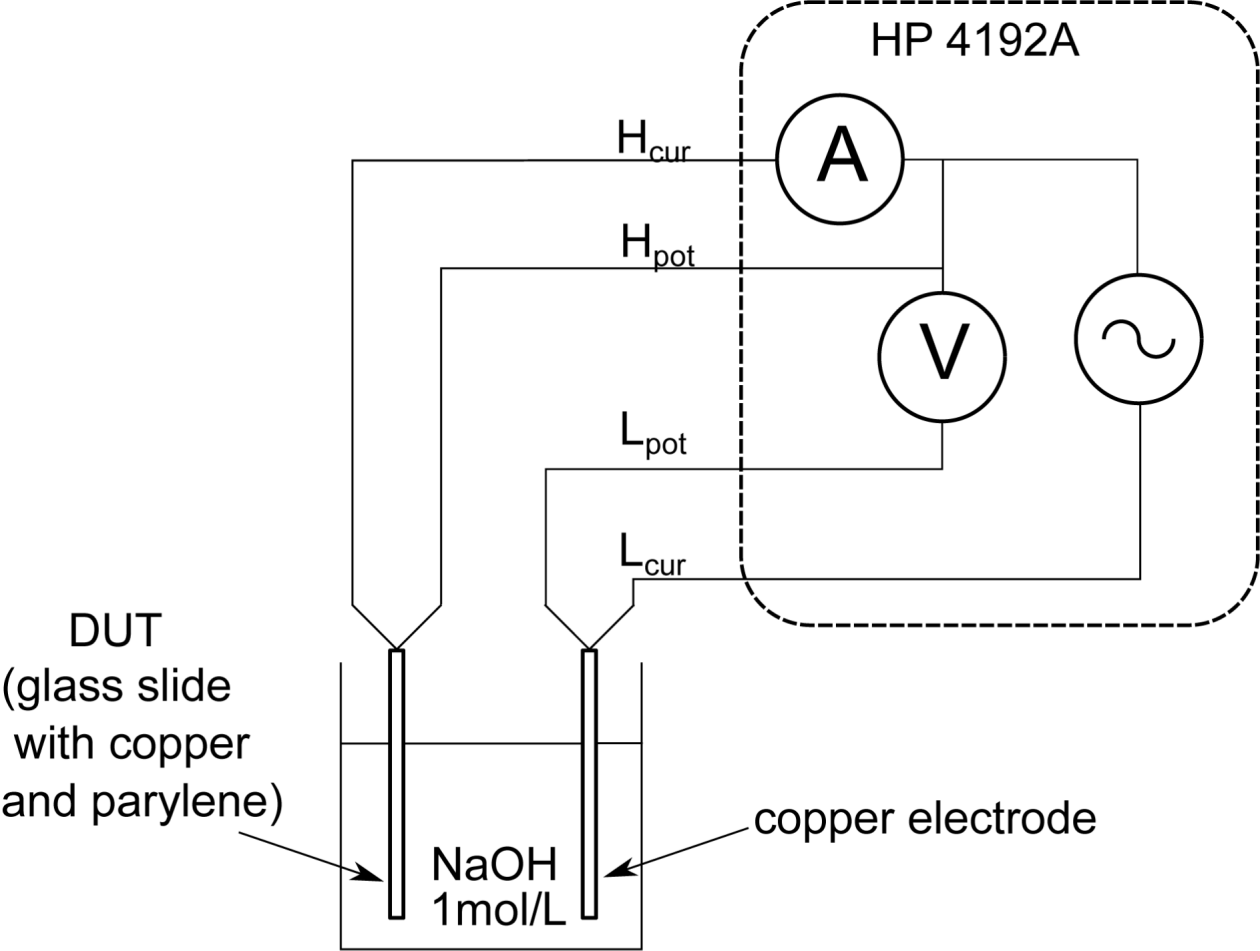
Experimental setup for electrochemical impedance spectroscopy: Between two copper electrodes in an aqueous solution of an electrolyte, here 1 mol/L NaOH, one coated with a porous layer (Device Under Test, DUT), a *I*(*V*) measurement can be carried out, which yields the frequency-dependent impedance. To get a precise measurement, a four-probe device is required (*H*_cur_ and *L*_cur_ denote the current terminals, *H*_pot_ and *L*_pot_ the voltage terminals). Reprinted with permission from [39], copyright 2012 American Vacuum Society.

To measure the impedance of the system the electrodes are connected to a HP 4192A Impedance Analyzer, which measures the impedance *Z* and the phase angle φ between test voltage and resulting current. The HP 4192A Impedance Analyzer (Hewlett-Packard, Palo Alto, Calif., U.S.A.) is able to sweep the frequency from a fraction of a Hertz up to 12 MHz (Figure 12).

This measurement is the basis for the determination of the frequency-dependent capacitance, which is obtained with the same measurement setup as in Figure 12. The measurement frequency was 1 MHz to minimize the influence of the Warburg capacitance. As the dimensions of the glass slide are known (area, film thickness), the permittivity ε was calculated according to Equation 3:

[3]
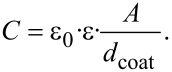


### Sandwich layer silver + PPX

#### Analysis

To investigate the antibacterial impact, two data sets have to be compiled, the time-dependent release rate of Ag^+^ ions, and its effect on bacteria. Several standard methods are in use to measure the released concentration of Ag^+^ ions, always at the detection limit. Among the physical and chemical standard methods to detect traces are inductively coupled plasma optical emission spectroscopy (ICP-OES), cyclic voltammetry, and atomic absorption spectroscopy (AAS). The former two methods have been extensively used and are described in [19,40–41].

The biological methods are the determination of the zone of inhibition around a spot of the subjected material, and the measurement of the optical density as a function of the time, i.e., absorption spectroscopy at a fixed wavelength (mostly used OD_600_). After having exposed a colony of bacteria with a certain starting density against solutions of Ag^+^ of known concentration or against solutions with Ag^+^ ions releasing catheters, the raising absorption is caused by exponential bacteria growth, which eventually becomes saturated. With this method, however, a decision is not possible whether the bacteria are already dead or still alive but have lost their ability for proliferation (the difference between bactericidic and bacteriostatic character). With the measured Ag^+^ concentrations, the minimum inhibitory concentration (MIC) against certain bacteria in certain solvents is established. In this article, we focus on the latter method because it allows for quantitative conclusions.

The medium of choice is artificial urine because our catheter should be applied to the uriniferous system [41–42]. Although urine is mainly a solution of NaCl (approx. 0.1 M), no precipitation of AgCl occurs because urine always contains ammonia (which is the reaction product of urea and urease). Only by the complexing reaction of Ag^+^ and NH_3_ to [Ag(NH_3_)_2_]^+^, the amount of free Ag^+^ can be kept below the level that forces Ag^0^ into its oxidic state Ag^+^ even in an anaerobic environment (the oxygen content of fresh urine in the renal pelvis is still unknown because it was never studied [19]). Physiological sodium salt solution, for example, never passes the limit of the solubility product at concentrations that are typical for these drug-release systems.

**Release rate:** The coated substrates [two catheter pieces (outer diameter: 5.3 mm) with a length of 2 cm (13% of the length of a normal balloon catheter, inner diameter: 2.3 mm) and completely coated with silver] were subjected to equal amounts (2 mL) of artificial urine for defined exposure times in 50 mL tubes (37 °C, 120 rpm) in the incubator [43]. After 24 h of incubation, the catheters were placed in fresh artificial urine. After one week of additional incubation, catheters were placed again in fresh artificial urine for another three weeks. The silver concentration in all three samples was measured by ICP-OES using an ICP710-ES (Varian Medical Systems, Willich, Germany), which covers a range of typically more than five orders of magnitude [40]. This method has become standard for determining atomic concentrations in highly diluted solutions at the detection limit (5 μg/L = 50 nmol/L) [44]. Summing up all three values resulted in the total amount of silver released over a period of four weeks [45]. Additionally, voltammetry was used to measure the ionic concentration only [41].

**Antibacterial impact (optical density):** The sterile specimens were catheter pieces (outer diameter: 5.3 mm) or one catheter piece (outer diameter: 5.3 mm) of a length of 5 mm (inner diameter: 2.3 mm), completely coated with silver and coated with PPX layers of different thicknesses. The specimens were incubated in 1 mL of artificial urine for 24 h at 37 °C. After having removed the samples, 450 μL of the supernatant was transferred into a sterile 48-well plate and inoculated with 10^3^ CFU/mL of *E. coli* or *S. cohni*. Bacterial growth was measured photometrically at a wavelength of 600 nm every 20 min for 21 h (TECAN Infinite M200 PRO Nano Quant, Tecan Trading AG, Männedorf, Switzerland) [41].

## Results and Discussion

### Silver film

Absolute prerequisite is the hydrophilization of the surface. Bi et al. have shown by X-ray photoelectronic spectroscopy (XPS) that O_2_ treatment of PPX under similar exposure conditions causes the origin of two new peaks at 287.8 eV and 289.3 eV in the C *1s* spectrum, which they attributed to the carbon atoms in the free carbonyl group (C=O) and carbonate group (O_2_C=O), respectively [36].

**Figure 13:**
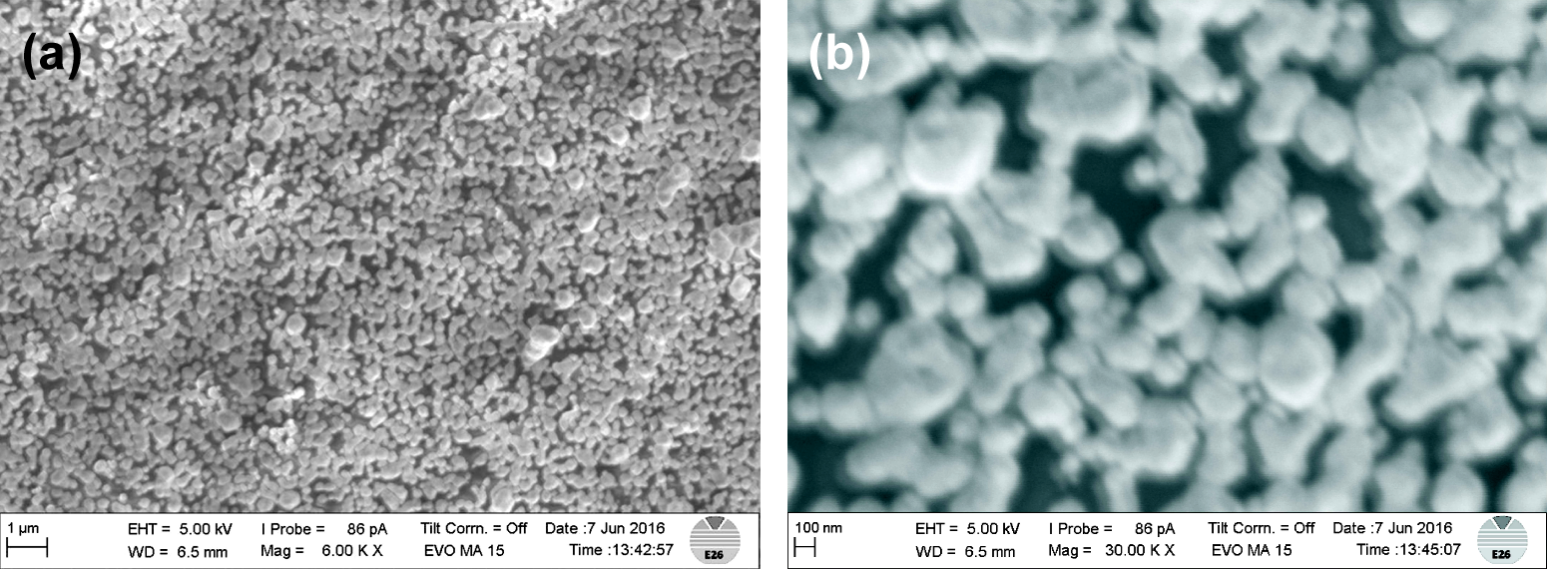
(a) The low-resolution SEM micrograph of silver on a surface of thermoplastic polyurethane (TPU) shows small adjacent grains of silver (5 min deposition time), saccharide: maltose. (b) The high-resolution SEM micrograph reveals a typical grain size between 50 and 200 nm.

On smooth substrates, the growth can be evaluated without any distortion. In Figure 13, silver grains on thermoplastic polyurethane are depicted.

**Figure 14:**
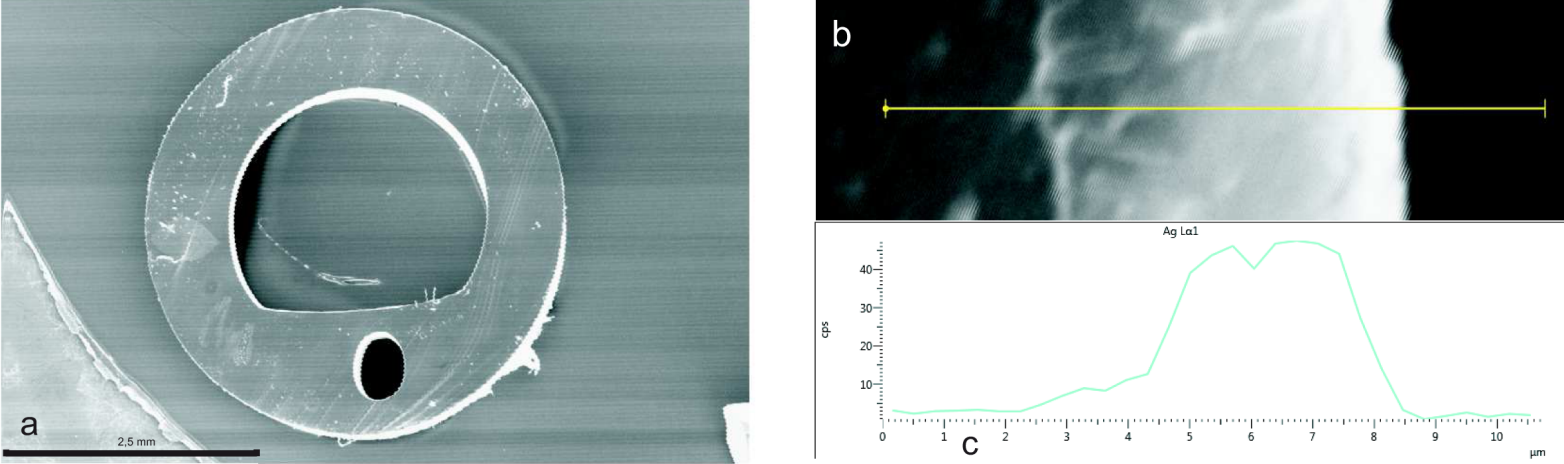
Silver layer deposited on the interior wall of a balloon catheter. (a) SEM micrograph of the cross section, (b) SEM micrograph of the silver layer, (c): EDX scan through the silver layer. Reprinted with permission from [19], copyright 2016 American Vacuum Society.

The surface is covered with adjacent silver grains, which are composed of small clusters with a typical diameter between 50 and 200 nm. Under the same reaction conditions but with longer exposure times, capillaries made of polysilicone were treated by Tollens’ reagens, and these thick layers have been subjected to SEM and EDX analysis (Figure 14). In Figure 14a, a cross section of an urethral catheter is displayed. Figure 14b shows the silver layer (*d* ≈ 4 μm) on the interior wall, and in Figure 14c, an EDX scan of the Ag Lα line along the yellow line in Figure 14b is shown.

As is evident from Figure 8, the thicknesses of the two layers, the silvery depot film and the polymeric cap layer, must match to avoid poor adhesion of the silver layer. Also, silver is exposed to an aggressive liquid that could passivate the reagent, thereby reducing the impact of retarded controlled release of Ag^+^ ions. An upper thickness limit of 500 nm is required to deliver an Ag^+^ concentration into the urine that suppresses the density of bacteria below a certain level, the minimum inhibitory concentration (MIC). This has been topic of our recent research [19,41]. However, for these low thicknesses, the Tollens’ reaction does not generate a continuous film on the rough surface of the catheters.

#### Morphology

**Figure 15:**
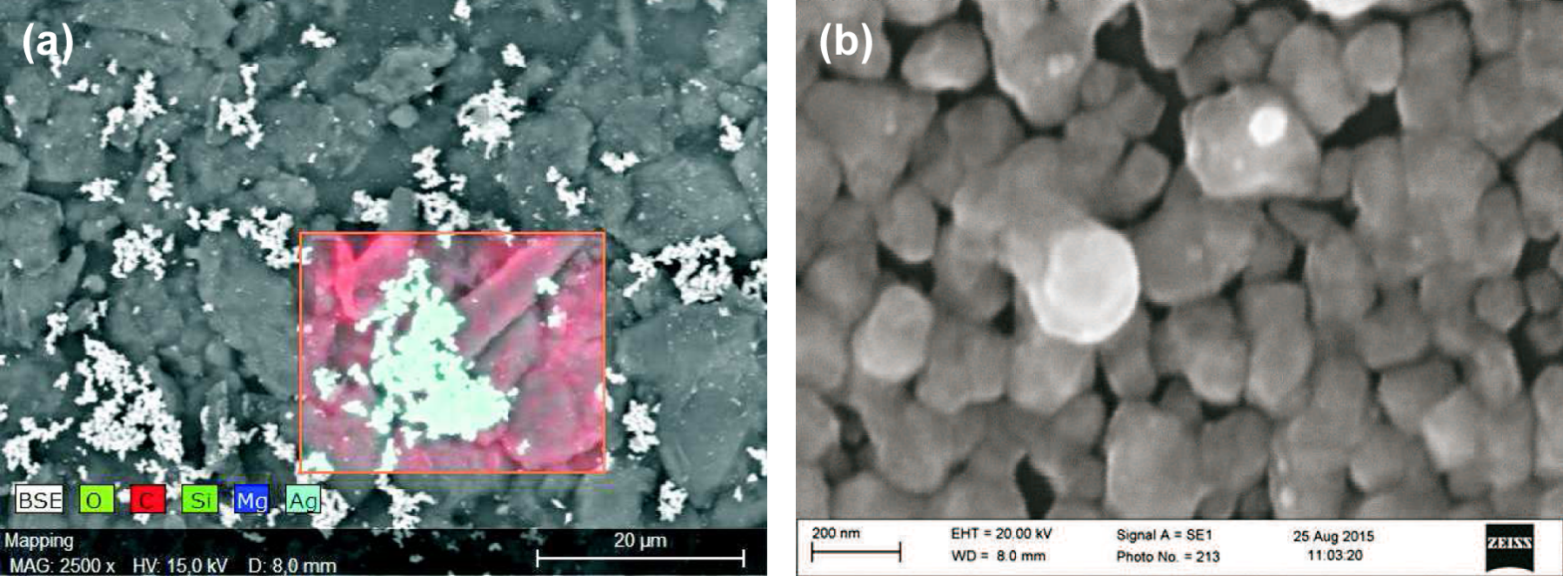
(a) The low-resolution SEM micrograph of silver on a rough polysilicone surface shows disconnected spots of silver (10 min deposition time, disaccharide: maltose). (b) The high-resolution SEM micrograph reveals a typical grain size of 50–200 nm. Reprinted with permission from [19], copyright 2016 American Vacuum Society.

In the following, we focus on these sub-micrometer layers on relatively rough surfaces. After a deposition time of 5–10 min, the resulting total silver layer on glass substrates exhibits a thickness between 200 and 500 nm. On polysilicone, however, incoherent spots of Ag are deposited, and the resulting grain size amounts to 50–200 nm (Figure 15). The grain size delicately depends on the pH value, on the type of sugar (monosaccharide or disaccharide), and to a lesser extent on the deposition time.

These micrographs justify these grains to be classified as nanoparticles [46–49]. Irrespective of how they have been generated, one of the main issues is their significantly enhanced surface, compared with thick coatings, although they have definitely passed the state of islands and have already coalesced [50]. This larger surface area should increase the release rate and therefore the antibacterial activity.

### PPX by CVD

#### Surface polymerization vs volume polymerization

The silver depot is capped by a layer, which acts in a threefold way: It should encase the silver grains completely and should touch the organic substrate. Only by meeting the first requirement, no direct access of the medium to the silver depot is ensured, which could cause uncontrolled solvation of Ag^+^ ions. By fulfilling the second demand, the adhesion of the grains is improved. But the main purpose of the cap layer is the exact control of the release rate of Ag^+^ ions. This is achieved by tuning the porosity of the cap layer as a function of the layer thickness.

Chemical vapor deposition differs from physical vapor deposition by the fact that one or more substances are evaporated and undergo a chemical reaction during transport to a surface. According to Figure 4, the radical polymerization reaction occurs at the two methylene groups in *para*-position of the benzene ring. The chain length can vary (molecular weights are typically in the 200000 to 400000 range [27,51–52]), and the reaction can occur in the vapor phase as well as during the process of condensation.

Therefore, the deposition must be steered into the direction of surface polymerization to avoid formation of larger clusters already in the vapor phase (volume polymerization). This problem was addressed for the first time by Yasuda et al. who diluted the chain-building vapor by an inert gas [53]. They found the expected reduced deposition rate. Additionally, they observed a vertical gradient from the vapor entrance to the pumping flange. According to them, the kinetic energy of the film-building species at substrate level had been reduced by collisions with the atoms of the inert gas [54].

The existence of these two competing processes in the volume and at the surface is a main issue in epitaxy, and the transition from simple chemical vapor deposition to advanced epitactic layer formation can be managed only by pushing back reactions in the vapor phase [55]. This can be easily achieved by dilution of the layer-forming gas(es) with an inert gas. In our case, the exact control of the porosity is mandatory and diluting the gases leads to high-quality, homogeneous layers with a high conformity, albeit at slow growth rates [39].

#### Growth rate as function of pressure

The reactor spatially separates the regions of activation (cleavage of dimers) and polymerization (oligomers or polymers in the vapor vs deposition as surface reaction). The growth rate, GR, depends on two parameters: the availability or density of monomers and on a steric factor. Two monomers are generated by the homogeneous fission of the dimeric precursor in the cracking unit. For the geometry of this reactor, a complete turnover to monomers is expected.

For the dependence on pressure, several experimental findings differ between an exponent of 1.5 [33] and 2 [56]. Models for the polymerization have been developed by Ganguli [33], Beach [57] and Fortin [58]. For a surface reaction, the diffusion of reactive species to an active site plays an important role. After the initial reaction of three monomers, which form a triple unit with two reactive centers, chain growth is proportional to the density of the monomers and the diffusivity of the monomer in the film. This leads to an exponent of only 3/2 for the overall pressure dependence [33,57]. It is remarkable that in the small pressure interval covered, the experimental data can be fitted well by both approaches.

At low vapor densities, surface polymerization is favored, and if the pressure is raised volume polymerization will predominate. This behavior defines an upper limit of the operating pressure due to the onset of parasitic snow formation (approx. 100 mTorr) [33].

It should be noted that the reaction is not diffusion-controlled [59]. The rate at which the radicals strike the surface was estimated to exceed that at which a radical is effectively absorbed by the growing chain by approximately three orders of magnitude [57]. This low sticking coefficient is mandatory for the excellent conformal coating, and the rate-limiting step happens on the surface.

**Figure 16:**
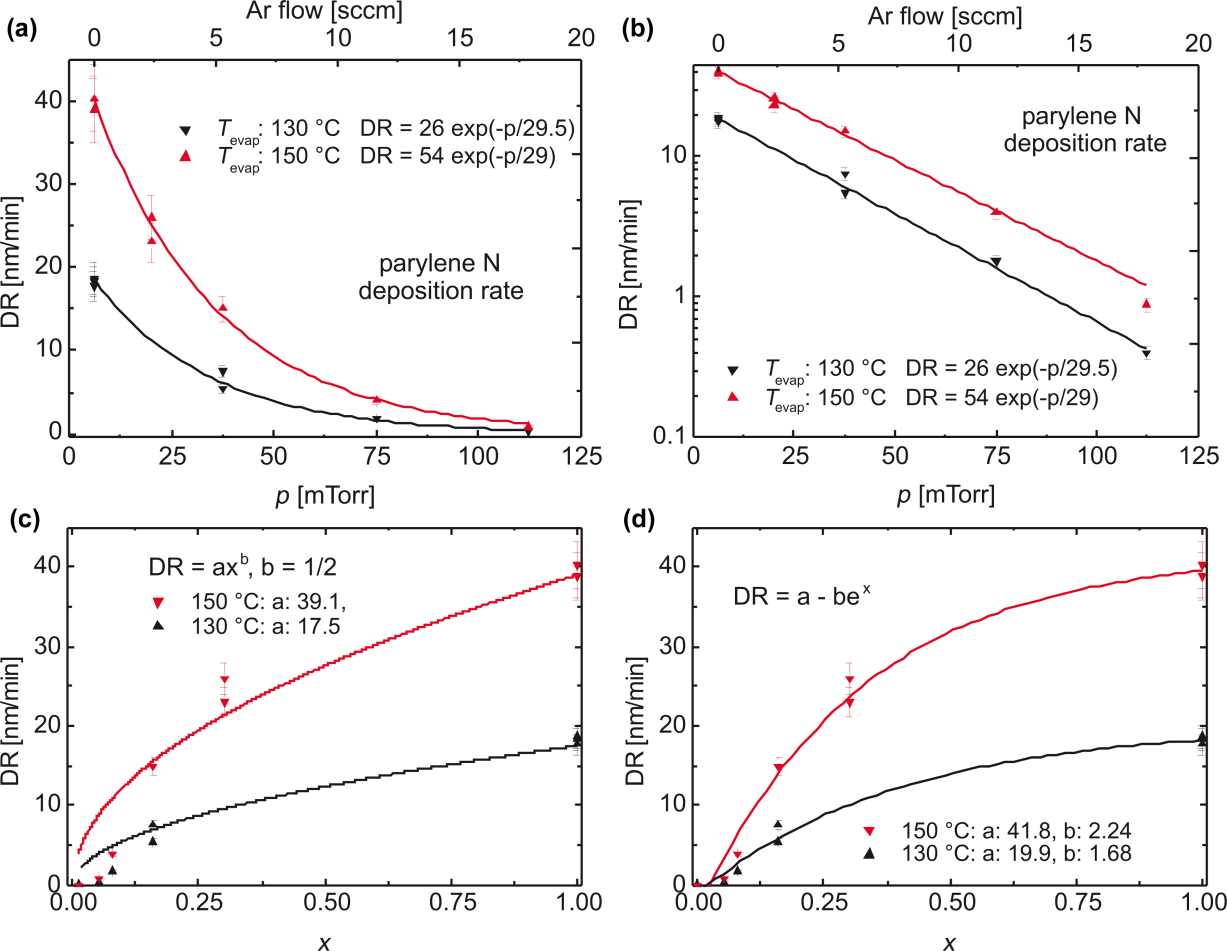
(a,b) Deposition rate of parylene-N as a function of the reactor pressure; (c,d) deposition rate of parylene-N as a function of the percentage of the partial pressure of parylene-N *p*_p_ = *f*_PX_/(*f*_PX_ + *f*_Ar_) for two models. By diluting the vapor with argon, the reaction is forced to surface polymerization (reaction of first order), best visible by the linear logarithmic slope [60]. In the model in panel c, the rate-limiting step is the condensation of the monomer; in the Beach model in panel (d) surface diffusion is the rate-limiting step.

As our interest is focused on layers with a defined porosity, which can only be achieved by a low deposition rate, diluting the chain-building vapor with an inert gas is one possibility to enhance the film quality. This is analyzed by variation of the evaporation temperature of the dimeric precursor (following the Clausius–Clapeyron equation), and diluting the reactive gas with argon. In Figure 16, the deposition rate is given as function of the total pressure (Figure 16a: linear scale and Figure 16b: logarithmic scale) and as function of the percentage of the partial pressure of the monomer for two models. The equilibrium constant between monomer PX and dimer DPX (Figure 4) is given by Equation 4

[4]
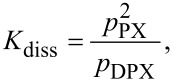


which is followed by the (intended) surface reaction PX→PPX. The equilibrium is written inverted (as in the derivation of the solubility product, Equation 5):

[5]
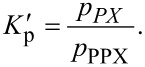


Because PPX is a solid with an activity of 1, it is integrated into the equilibrium constant *K*_p_ yielding Equation 6, which expresses diffusion control

[6]
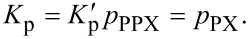


The condensation is followed by a chemical reaction (enlargement of the polymeric chain) with quantitative yield. Including Equation 4, this leads to the final Equation 7:

[7]
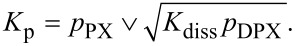


This dependence is shown in Figure 16c. Figure 16d shows the model of Beach, which connects the terminating value for PPX, here denoted *a*, with an exponential term *c^x^* and a pre-exponential factor *b*. *b* describes mainly the surface diffusivity, and e*^x^* includes the order of the surface diffusion reaction with its diffusion coefficient [57]. Evidently the experimental data can be fitted with that model.

#### Morphology (grain size)

**Figure 17:**
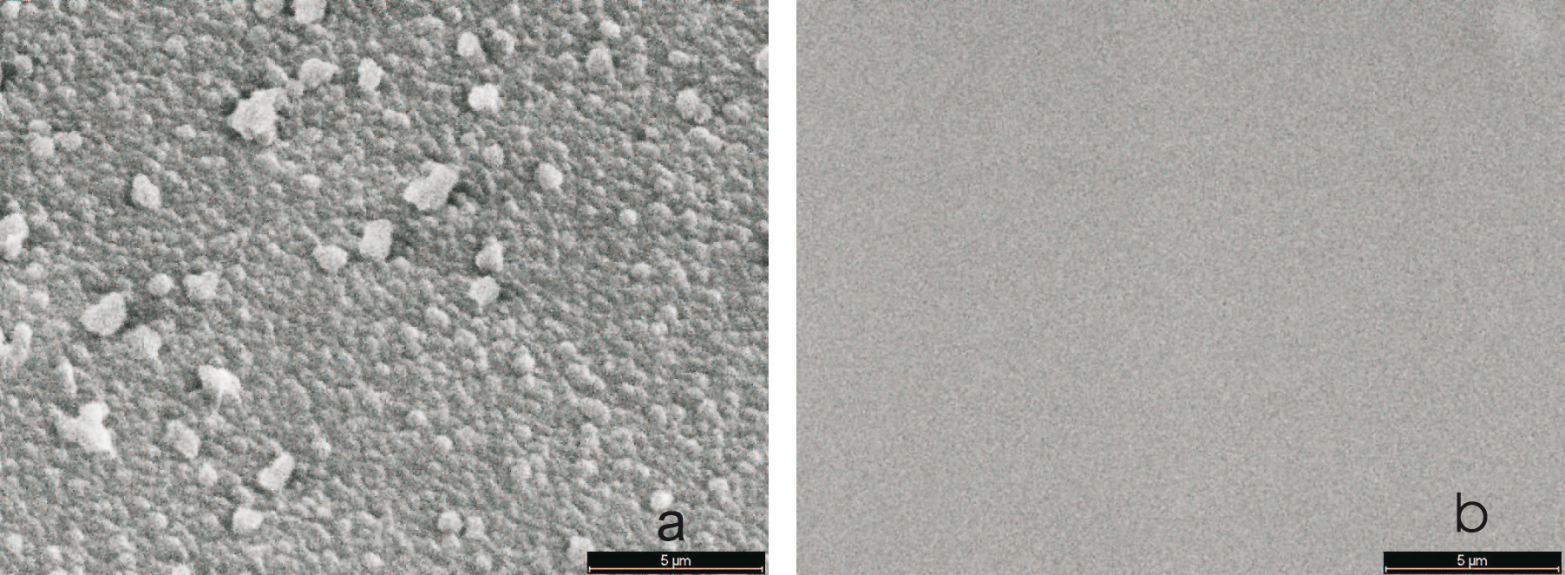
(a) Parylene N deposited in pure atmosphere of *p*-xylylene at a total pressure of 6 mTorr; (b) parylene N deposited in *p*-xylylene diluted with at a total pressure of 50 mTorr. At constant partial pressure of the monomer, the volume polymerization at low pressure is effectively suppressed. Reprinted from [60], copyright 2015 the authors.

By diluting the chain-building vapor with argon, collisions between the monomers, which lead to unwanted reactions are reduced. These unwanted reactions lead to polymerization and grain formation in the gas phase. Eventually, these grains arrive at the surface, which is clearly revealed by SEM inspection (Figure 17a).

#### Properties of sub-micrometer layers

**Figure 18:**
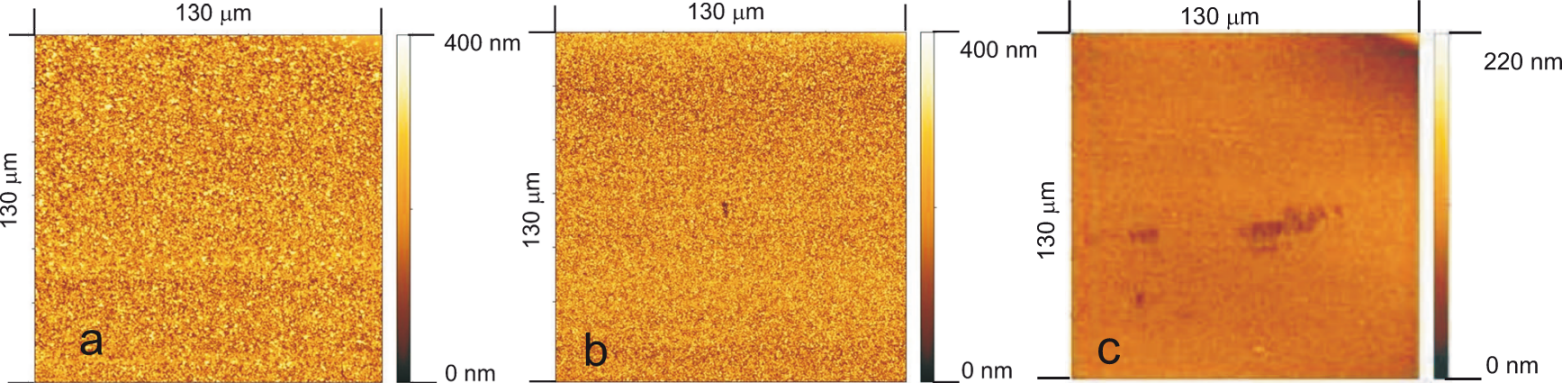
Surface morphology of parylene films deposited at various deposition pressures. Flow of the monomeric species was fixed to 9 sccm. Process pressure was established by adding argon to the ambient. (a) Without diluent, 6 mTorr (0.8 Pa), (b) 37.5 mTorr (5 Pa), (c) 75 mTorr (10 Pa). Reprinted from [60], copyright 2015 the authors.

As our interest is focused on very thin high-quality layers, visual inspection via SEM is only one method, which has to be flanked by electrical methods. AFM micrographs are shown across an area of 130 μm × 130 μm in Figure 18. Again, increasing smoothness of the film with increasing dilution with argon is shown.

**Figure 19:**
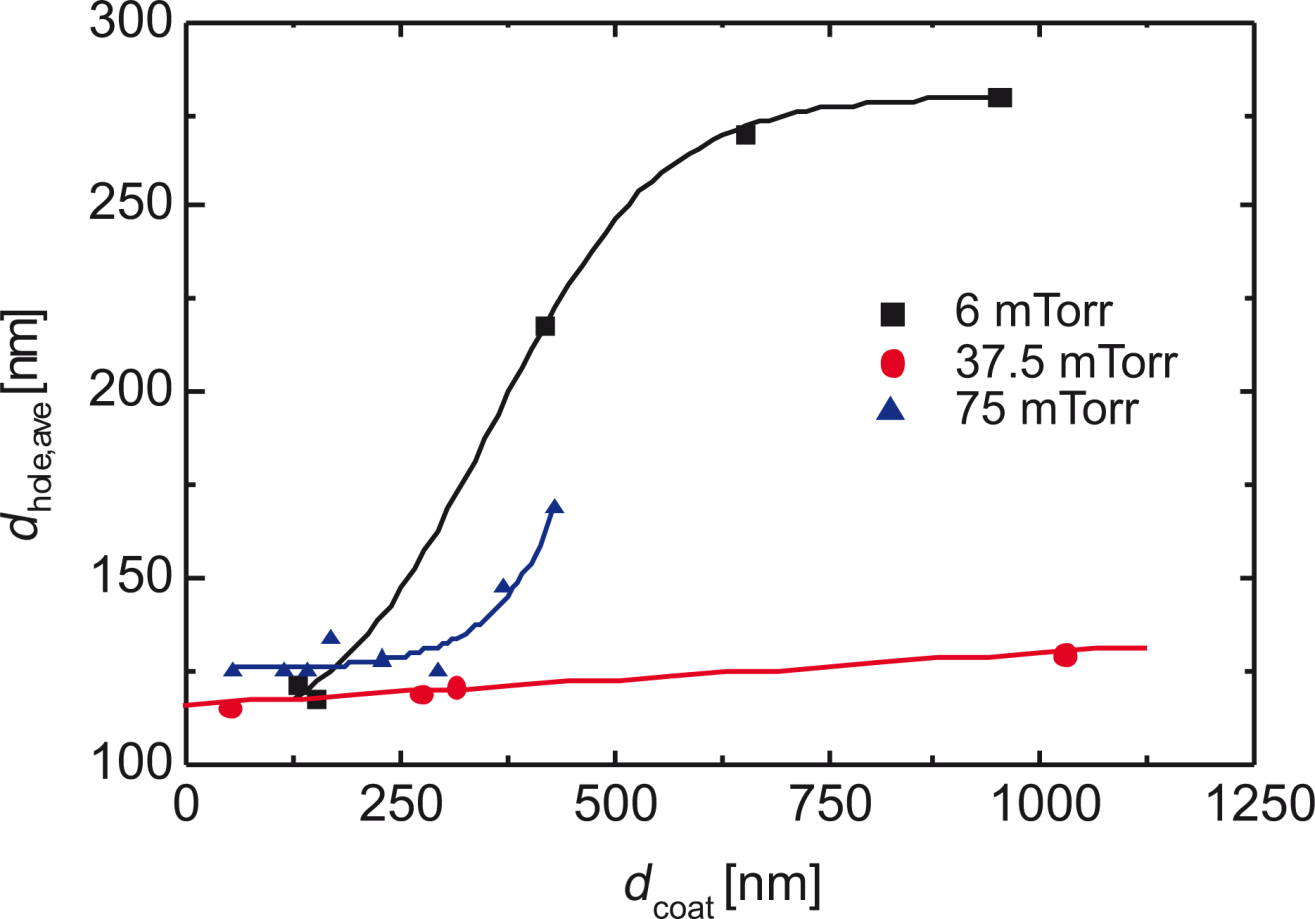
Averaged pin hole density of PPX-N at 90% scan depth of AFM [61].

Evaluating the AFM micrographs with GWYDDION reveals the imperative necessity to dilute the monomer by an inert gas (Figure 19). In pure monomeric vapor, the hole density increases steeply but almost uncontrollably.

**Figure 20:**
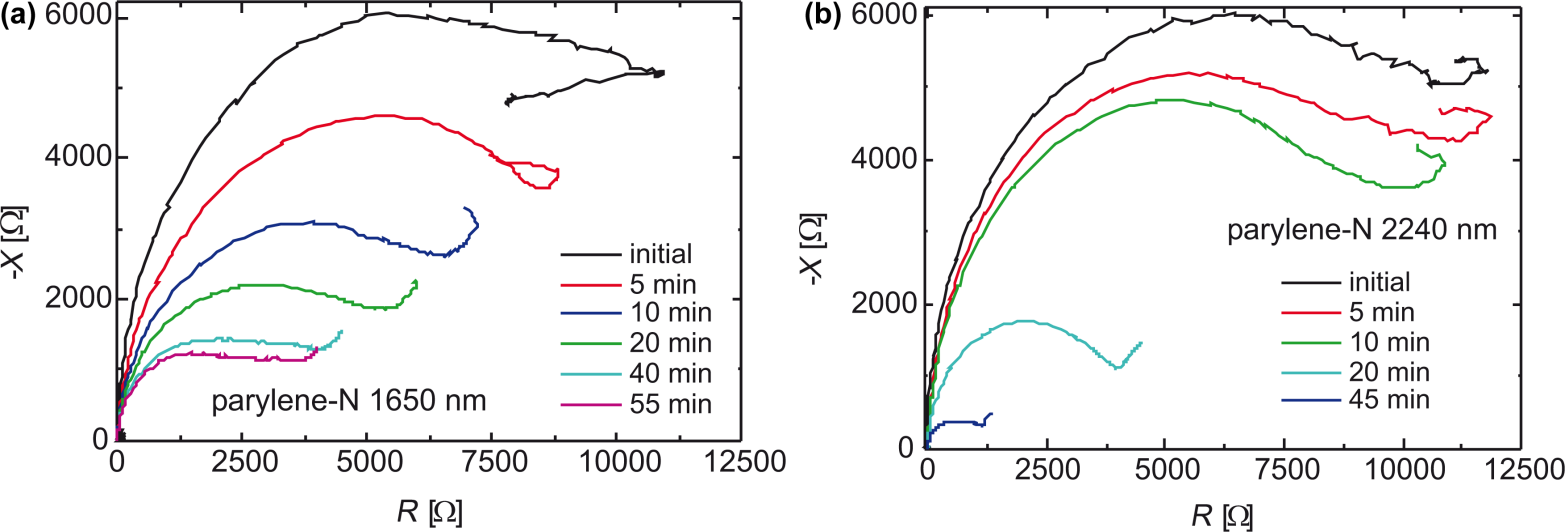
Nyquist diagrams of an metallic electrode covered by a porous membrane of PPX exhibiting a thickness of (a) 1650 nm and (b) 2240 nm. Imaginary part *−X* as a function of the real part *R* of the impedance. Reprinted with permission from [39], copyright 2012 American Vacuum Society.

The EIS measurement results are shown in a Nyquist diagram (Figure 20), where the device under test was a copper layer, which had been coated by PPX-N (1650 and 2240 nm).

According to the equivalent circuit in Figure 11, the intersection of the graph with the real axis close to the origin is related to the serial ohmic resistance *R*_Ω_ of the NaOH solution. The semi-circle trajectory, which approaches the shape of a straight line for lower measurement frequencies, is caused by the capacitance *C*_dl_ of the NaOH–PPX–Cu double layer. The slope of the straight line is caused by the so called Warburg *Z*_W_ impedance.

Without the influence of *Z*_W_, the half-circle would intersect the abscissa for low frequencies at *R*_f_ + *R*_Ω_, since *R*_f_ represents the ohmic behavior of the coating. The local minimum at Re(*Z*) = 3250 Ω is caused by the measuring frequency of 100 Hz. Hence, all capacitance measurements of the PPX layer have to be carried out at freqencies significantly above 100 Hz to avoid confusing *C*_dl_ and *Z*_W_. Because the silver ions were expected to move slowly in the porous membrane, the measurement cycle was repeated every 5 min.

**Figure 21:**
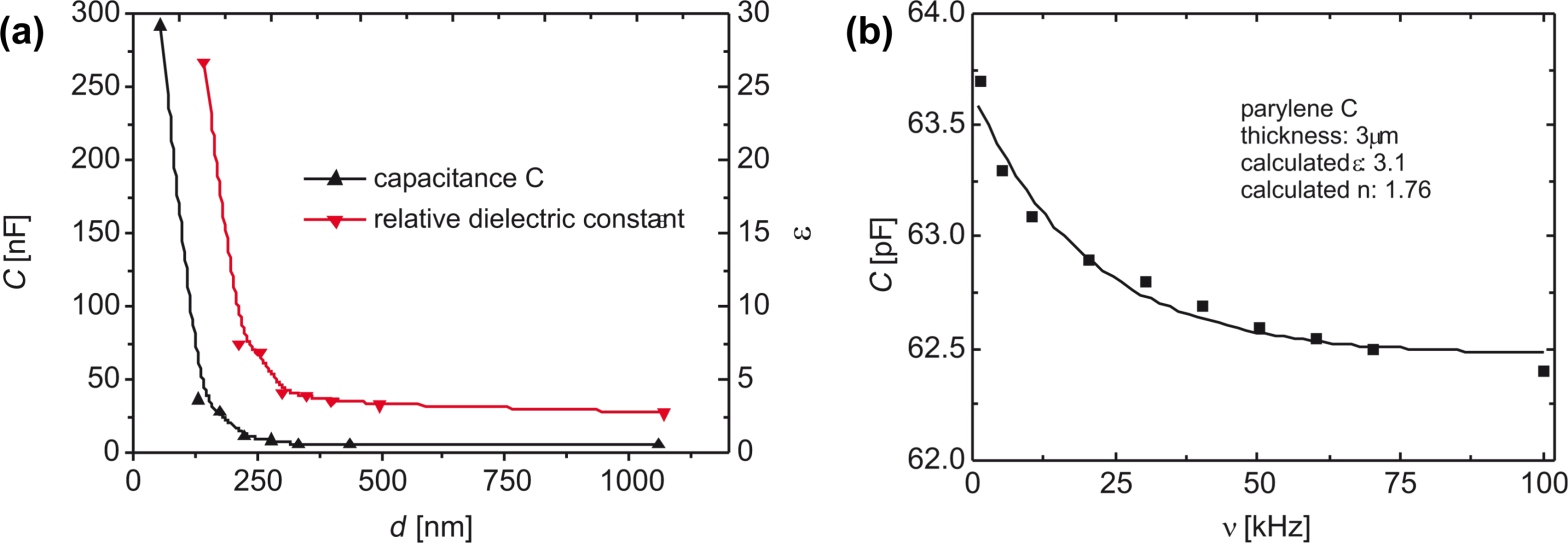
(a) Areal capacitance of CVD layers of PPX-C and resulting permittivity as function of thickness as measured with electrochemical impedance spectroscopy at 1 MHz. (b) Capacitance of CVD layers of PPX-C and resulting permittivity as function of frequency in the low-frequency regime. Reprinted with permission from [39], copyright 2012 American Vacuum Society.

Applying electrochemical impedance spectroscopy, the areal capacitance of layers of PPX-C is measured and ε is calculated with Equation 3. The striking increase below a thickness of 350 nm is caused by the increasing porosity. For thicker layers, ε reaches its limit of 3 (literature value at 1 MHz: 2.95 [62], Figure 21a). This value matches perfectly the low-frequency capacitance. In Figure 21b, the capacitance as function of frequency is shown in the low-frequency regime up to 100 kHz. The value starts at an almost constant bottom level of 3.1 at 100 kHz to rise to lower frequency values (literature value at 1 kHz: 3.10 [62]).

#### Deposition of layers with longitudinally uniform thickness

From Equation 1 and Equation 2, it has become evident that deposition on the interior of a long pipe is inevitably connected with a steep gradient in layer thickness. The simple approach of these equations does not take into account a steric factor: The probability of a radical to be caught at the terminal position of the polymeric chain is assumed to decrease strongly with the growing length of the chain. This leads to a complicated dependence, which was extensively discussed by Broer and Luijks, and Tolstopyatov et al. [63–65].

**Figure 22:**
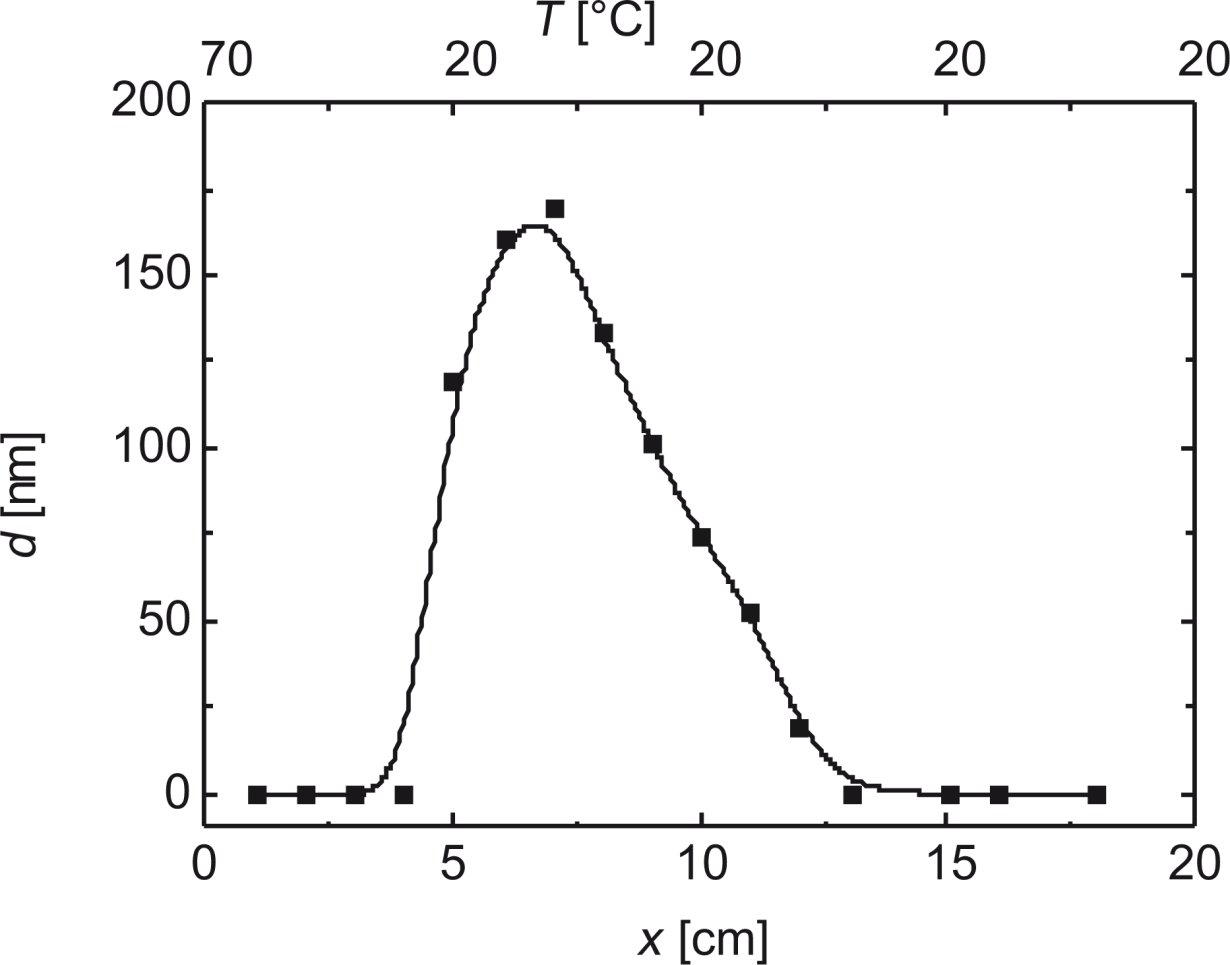
The maximum in deposition rate is moved to the center of the capillary by establishing an appropriate temperature gradient. The figures at the top denote the temperature of the Peltier elements.

This steep thickness gradient is fought by the application of Le Chatelier’s principle. The principal sketch of the temperature seesaw with two Peltier elements along the groove in which the pipe is embedded takes the exothermal principle of the condensation reaction into account (cf. Figure 7). A higher temperature at the opening of the pipe prevents condensation and the subsequent polymerization. Hence, it should be possible to shift the maximum in deposition rate towards the center of the capillary by establishing an appropriate temperature gradient. In Figure 22, the deposition maximum is moved from the opening of the capillary 5 cm towards the inside.

**Figure 23:**
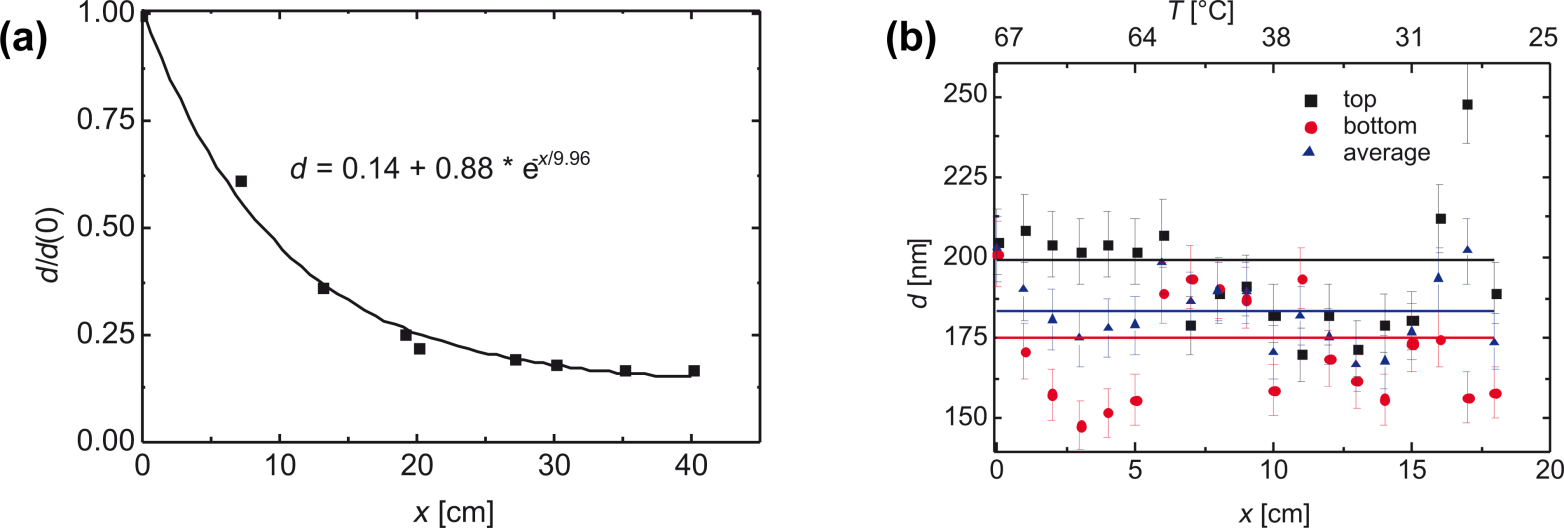
(a) Relative layer thickness (referred to the starting value at the mouth) as a function of the capillary length without a temperature gradient. (b) Layer thickness as a function of the capillary length after application of a counteracting temperature profile [66], error bars are ±5%. Reprinted with permission from [35], copyright 2017 American Institute of Physics.

Our construction of a temperature seesaw with five Peltier elements establishes a very effective temperature gradient, which eliminates the concentration gradient. Thus, the layer thickness is almost constant between opening and closed end of the capillary, at the cost of reduced deposition rate. This gradient is easy to apply at atmospheric pressure, but turns out to be difficult to control in vacuum. In Figure 23, a comparison is carried out of dependence of the layer thickness along a capillary without and with temperature balance.

### Thin coatings as porous membranes

During a parallel work, the minimum inhibitory concentration of Ag^+^ ions against the bacterium *E. coli*, which is responsible for approximately 80% of the nosocomial infections [1], was evaluated up to 30 μg/L in artificial urine measuring the optical density [19,41].

Although it is widely accepted that only Ag^+^ ions are soluble in water and can pass through the channels of the porous cap layer, it might be possible that also very small clusters of metallic silver could succeed in moving through the barrier [67]. Whereas with ICP-OES, it is not possible to distinguish between Ag^0^ and Ag^+^, because both species are excited by an inductively coupled plasma, with voltammetry, only ions are detected.

**Figure 24:**
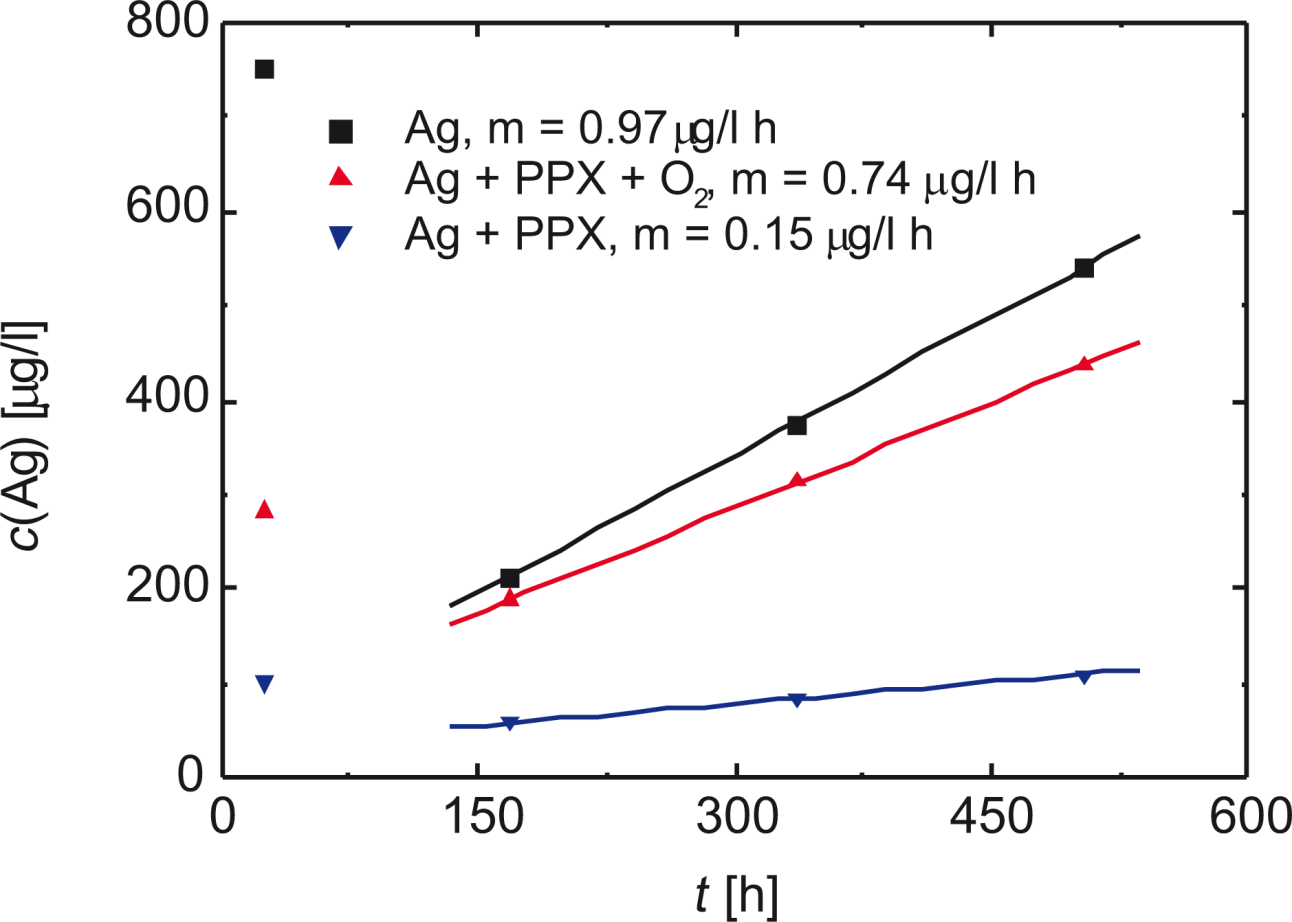
After 24 h, the released amount of Ag^+^ ions from the PPX layer does not depend on time and is almost constant. Reprinted with permission from [19], copyright 2016 American Vacuum Society.

In Figure 24, the silver release as measured by OES is depicted for an exposure of the sandwich layer against artificial urine for four different times: 24 h, 7 days, 14 days, and 21 days [68]. The release from the PPX layer is almost constant over time. Note that for this experiment, two pieces of a fully coated catheter were used. Their total length equaled only 13% of a complete balloon catheter (12”).

With these measurements, it could be shown that the desired thickness of the cap layer is between 150 and 300 nm [40], which, in turn, determines the upper limit for the thickness of the silver depot. In fact, the mean value of 50 μg/L or 0.45 μmol/L corresponds exactly to the results, which are possible in an environment that is determined by the presence of Cl^−^, but also of urea and urease. Small amounts of Ag^+^ ions are soluble in artificial urine as complexed [Ag(NH_3_)_2_]^+^, and the value is always above the MIC of 30 μg/L. [19].

### Antibacterial activity

**Figure 25:**
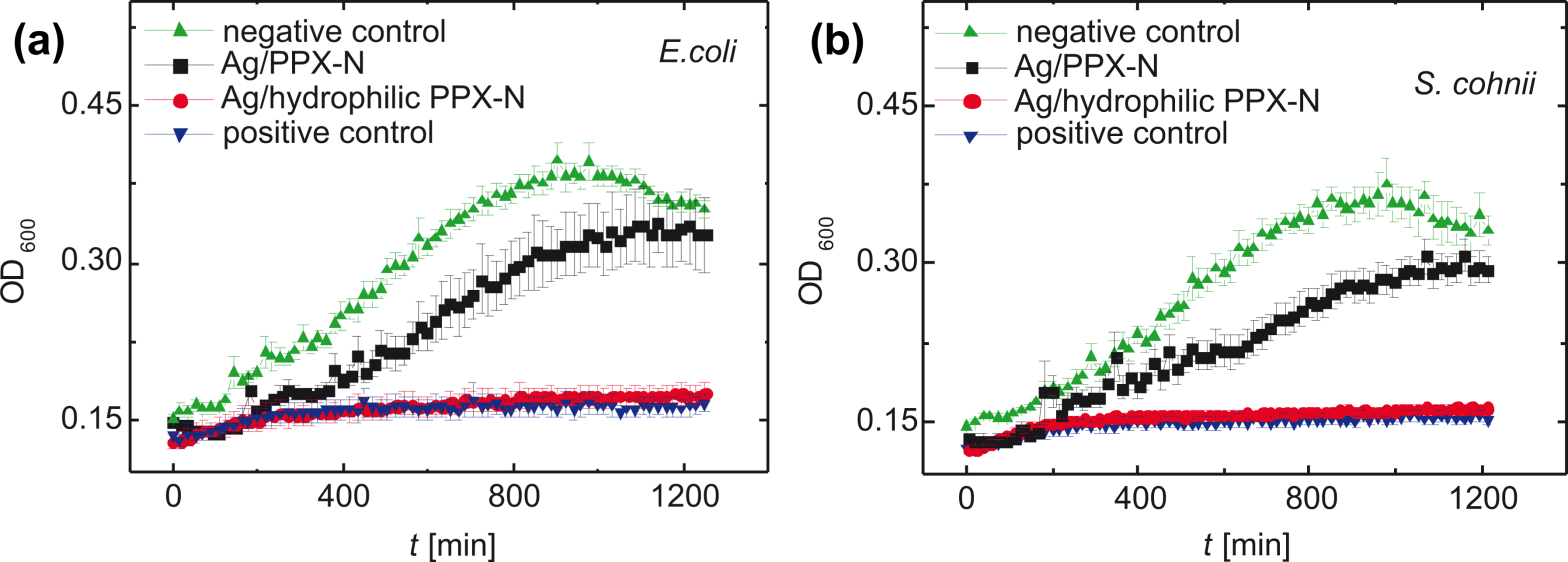
Growth of (a) *E. coli* and (b) *S. cohnii* in artificial urine, exposed to a defined catheter area with two different antibacterial sandwich layers. Positive control with untreated catheter and penicilline–streptomycine, negative control with untreated catheter. Optical density as a function of the exposure time. Urine was inoculated with 10^3^ CFU/mL, *n* = 6, *p <* 10^−4^ (ANOVA). Reprinted with permission from [41], copyright 2017 American Vacuum Society.

Measuring the optical density is a preferred method to determine quantitatively the bactericidic power of a reagent as function of time. One can distinguish the lag phase at the begin of the treatment when the bacteria adopt to the new environment, which causes a very low division rate. It is followed by the exponential growth phase, leading to typical log values of 0.4–0.6. A further increase is suppressed by the increasing competition for nutrition, which leads to the plateau phase and eventually to a darkening of the medium. Although this effect is known to be caused by a large number of dead bacteria, a distinction between active cells and those who are either still alive but not capable to divide themselves or are already dead is impossible. In the case of successful antibacterial treatment, no increase in optical density is expected, and the antibacterial activity can be quantitatively evaluated (Figure 25).

## Conclusion

One of the most underestimated issues in clinical treatment is the ubiquitousness of aggressive bacteria that cause thousands of victims after a successful surgery. Urological inflammations are responsible for approximately one quarter of all nosocomial infections. These infections are most often attributed to the balloon catheter, which has been implanted to more than three million patients in Germany in 2015. Since many bacteria have developed a highly effective resistance against most antibiotics, other strategies are highly welcome, strategies that act on-site.

The construction and fabrication of a new antibacterial system, which belongs to the category of drug-release devices, has been described. Starting with commercially available catheters, its main feature is a layered sandwich coating, which is composed of a fragmented base layer of silver capped by a thin film of poly(*p*-xylylene). This top layer is designed to release a controlled current of Ag^+^ ions. With this feature, it is possible to tune their concentration to a level, which is bacteriostatic, but well below the toxic level for humans.

For an effective protection, this sandwich layer has to be deposited on the interior and the exterior side of the capillary. Coatings out of the liquid phase are always possible, provided that the surface is sufficiently wetted, but most vacuum-based methods fail to coat the inner surface (sputtering, evaporation), only vapor phase deposition is an appropriate technique.

With these restrictions in mind, the recipe for this microsystem has been elaborated. The base layer has been deposited electrolessly applying Tollens’ reagent, the cap layer has been coated using chemical vapor deposition. The three main problems of this process, which are electroless coating of a hydrophobic substrate with a silver layer with an aqueous solution of a silver salt, irreproducible evaporation during heating of the precursor, and exponential decrease of the layer thickness along the capillary, have been solved by application of three standard principles of chemistry and physics: electrochemical reactions applying simple redox equations, Papin’s pot and the principle of Le Chatelier.

These sub-micrometer layers are permeable for Ag^+^ ions and small Ag^0^ clusters. The diffusion coefficient can be tuned by the thickness of this film. The sandwich system acts antimicrobially against conventional bacteria on-site and avoids the application of antibiotics by substituting them by oligodynamic silver.

This sandwich technique of a drug-releasing system has been realized with a device, which is a challenge for the coating technology due to its high aspect ratio. With the same approach, a perforated tube, e.g., a coronary stent, could be modified far more easily to become a “real” restenotic stent. The critical issue here is the roughening of a smooth surface to enhance its area for a higher load of an antistenotic drug. Increasing the aspect ratio, which is the case for urethral stents (1 mm open lumen at 150–200 mm in length), requires to overcome increased difficulties during coating. Such works are currently ongoing.
